# Spuriously transcribed RNAs from CRISPR-sgRNA expression plasmids scaffold biomolecular condensate formation and hamper accurate genomic imaging

**DOI:** 10.1093/nar/gkaf192

**Published:** 2025-03-22

**Authors:** Shiqi Mao, Ruonan Wu, Weibang Luo, Jinshan Qin, Antony K Chen

**Affiliations:** Department of Biomedical Engineering, College of Future Technology, Peking University, Beijing 100871, China; Department of Biomedical Engineering, College of Future Technology, Peking University, Beijing 100871, China; Department of Biomedical Engineering, College of Future Technology, Peking University, Beijing 100871, China; Department of Biomedical Engineering, College of Future Technology, Peking University, Beijing 100871, China; Department of Biomedical Engineering, College of Future Technology, Peking University, Beijing 100871, China; Beijing Advanced Center of RNA Biology (BEACON), Peking University, Beijing 100871, China; National Biomedical Imaging Center, Peking University, Beijing 100871, China

## Abstract

Clustered regularly interspaced short palindromic repeats (CRISPR)-based imaging tools that utilize fluorescently tagged single-guide RNAs (sgRNAs) have enabled versatile analysis of the dynamics of single genomic loci, but the accuracy may be hindered by nonspecific subnuclear probe accumulation, generating false-positive foci in cell nuclei. By examining the subcellular localizations of sgRNA expression plasmids, their RNA transcripts, and several RNA-binding proteins, we found that spuriously transcribed (cryptic) transcripts, produced by sgRNA expression plasmids, are the major contributors of false-positive signals, independent of sgRNA scaffold design or effector probe (i.e. RNA aptamer- or oligonucleotide-based probes) used. These transcripts interact with the paraspeckle core proteins, but not with the sgRNA expression plasmids or the paraspeckle RNA scaffold NEAT1_2, to form nuclear bodies that display liquid-like properties including sphericality, fusion competence, and sensitivity to 1,6-hexanediol. Transfecting sgRNA transcription units (i.e. sgRNA expression cassettes), lacking the plasmid backbones, reduces false-positive signals and enhances genomic imaging accuracy. Overall, this study unveils previously undescribed activities of cryptic plasmid transcripts and presents an easy-to-adapt strategy that can potentially improve the precision of CRISPR-based imaging systems that implement fluorescently tagged sgRNAs.

## Graphical Abstract

**Graphical Abstract:**
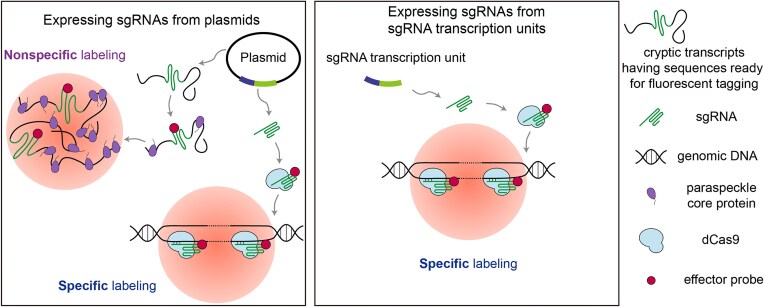

## Introduction

The clustered regularly interspaced short palindromic repeats (CRISPR)/CRISPR-associated protein 9 (CRISPR/Cas9) gene editing system has been repurposed to realize live-cell genomic imaging, achieved by inactivating the protein's nuclease activity (dCas9) without abolishing single-guide RNA (sgRNA)-mediated DNA binding [1]. To light up a specific locus, either dCas9 or sgRNA has been tagged with fluorogens, and sufficient tagging may render the target locus detectable as a discrete bright spot under fluorescence microscopy [2–4]. Notably, in studies where more sensitive imaging is required, the sgRNA-tagging approach appears to be a preferred choice. This is in part because unbound fluorescently tagged Cas9 proteins can display high background nuclear staining as a result of specific binding with nuclear proteins such as ILF3 and NCL, or with cellular RNAs that have similar sequences or structural features as sgRNA [5–8]. Additionally, because of its highly cationic nature, Cas9 may bind electrostatically to genomic DNA, independent of sgRNA [9–12]. Moreover, Cas9 is less amenable to modifications compared with sgRNA, which possesses tetraloop, stem-loop 2, and 3′ tail regions that are highly adaptable for fluorescent tagging by fluorescent proteins or organic dyes [2].

Despite the growing use, we and others have previously reported that fluorescently tagged sgRNAs, regardless of whether they are designed to target a nonsense or a specific genomic sequence, can accumulate nonspecifically in the nucleoplasm, forming discrete bright foci that can be mis-interpreted as authentic genomic loci [13, 14]. No such foci were reported when cells were instead transfected with synthetic guide RNAs [7], suggesting that formation of false-positive foci is solely a plasmid-dependent process. To date, the nature of plasmid-mediated false-positive foci formation has remained elusive, although these foci have been speculated to arise from accumulation of nascent sgRNA transcripts around their producer plasmids [14]. This is based on the observation that different sgRNAs transcribed from a single plasmid can form more colocalized foci than when the sgRNAs were transcribed from separate plasmids.

Given that plasmid transfection is highly versatile and useful for introducing CRISPR sgRNAs into mammalian cells, particularly in multiplex imaging applications where different guide RNAs must be present in the same target cell [15], the present study aimed to investigate the mechanism underlying the plasmid-mediated false-positive foci formation. Specific tasks were performed to examine the subcellular localizations of the sgRNA producer plasmids, their RNA transcript products, and several RNA-binding proteins, in the presence or absence of dCas9. Our results indicated that false-positive foci do not result from nonspecific accumulation of sgRNAs as previously speculated. Instead, they pointed to a previously undescribed role of cryptic plasmid transcripts, which arise from spurious transcriptional activities occurring within plasmid DNA regions other than the intended promoter-gene cassette (i.e. transcription unit) [16–19]. These unintended transcripts are sequestered by paraspeckle proteins FUS, SFPQ and PSPC1 rather than accumulating around their producer plasmids, leading to false-positive foci formation. Based on these findings, we presented an easy-to-adapt strategy to minimize the generation of false-positive signals.

## Materials and methods

### Plasmid construction

#### sgRNA encoding plasmids (for mammalian expression)

To generate pUC19-U6-sgNonsense that encodes the original sgRNA scaffold carrying a nonsense spacer (sgNonsense) (Supplementary Tables S1–S2), a backbone plasmid harboring a U6-sgNonsense cassette was custom-made by GENEWIZ, Inc. Thereafter, the PCR product of the U6-sgNonsense cassette from the backbone plasmid (Forward primer: 5′-ACTGCTGAATTCGATCCGACGCGCCATCTCTAGG-3′; reverse primer: 5′-ACCTGCAAGCTTAAAAAAAGCACCGACTCGGTGCCACTTT-3′) was inserted into the pUC19 vector (Takara Bio) digested with EcoRI and HindIII. pUC19-U6-sgNonsense-2XMS2, pUC19-U6-sgChr3q29_2–2XPP7, pUC19-U6-sgChr3q29_1-MTS*β*, and pUC19-U6-sgNonsense-MTS*α* were generated as described for pUC19-U6-sgNonsense (Supplementary Tables S1–S2). pUC19-CMV-sgNonsense was generated by inserting the PCR-derived CMV-sgNonsense cassette from a custom-made backbone plasmid (GENEWIZ, Inc) (Forward primer: 5′- ACTGCTGAATTCTGGAGTTCCGCGTTACATAACT-3′; reverse primer: 5′-ACCTGCAAGCTTAAAAAAAGCACCGACTCGGTGCCACTTT-3′) into the pUC19 vector digested with EcoRI and HindIII. To generate pGEM-U6-sgNonsense, the EcoRI- and HindIII- digested PCR product of the U6-sgNonsense cassette was inserted into the pGEM-11zf(+) vector. To generate pcDNA3.1/Hygro(+)-U6-sgNonsense, the U6-sgNonsense cassette was first obtained by restriction enzyme digestion of pUC19-U6-sgNonsense with EcoRI and HindIII. The excised fragment was inserted into the pcDNA3.1/Hygro(+)/CMV(-)/polyA(-) vector, which was created by PCR amplification of pcDNA3.1/Hygro(+) (Thermo Fisher) to remove sequences spanning the CMV region and the bGH poly(A) signal (forward primer 5′- TGGCACCGAGTCGGTGCTTTTTTTAAGCTTCTTCTGAGGCGGAAAGAACCAGCTG -3′; reverse primer: 5′-GGCCTAGAGATGGCGCGTCGGATCGAATTCAACGCGTATATCTGGCCCGTACATC -3′), using Gibson Assembly (Beijing Lablead Biotech Co., Ltd). pUC19-U6-sgNonsense_2, pUC19-U6-sgChr3q29_1–2XMS2, and pUC19-U6-sgChr3q29_2-MTS*α* were generated by PCR-mediated site-directed mutagenesis of the respective parental plasmids pUC19-U6-sgNonsense, pUC19-U6-sgNonsense-2XMS2, and pUC19-U6-sgNonsense-MTS*α* using primers listed in Supplementary Table S3.

#### sgRNA encoding plasmids (for *in vitro* transcription)

To generate pGEM-T7-sgCASFISH (Supplementary Tables S1–S2), a fragment containing the T7-sgCASFISH cassette was first created by PCR amplification of pSLQ1661-sgMUC4-E3(F + E), a gift from Dr. Bo Huang and Dr. Stanley Qi (Addgene plasmid #51025) [5], using forward primer 5′- ACTGCTGAATTCTAATACGACTCACTATAGGGCTTGAAAAAGTGGCACCGAGTGTTTAAGAGCTATGCTGGAAACAGCA-3′ and reverse primer 5′-ACCTGCGTCGACATCTGACGGTTCACTAAACC-3′. The PCR product was then inserted into a EcoRI/SalI-digested modified pGEM-11zf(+)-based vector that was created by PCR amplification of the original pGEM-11zf(+) plasmid using forward primer 5′-ACTGCTGTCGACTACTAGGATCCGGGCCCTCT-3′ and reverse primer 5′- ACCTGCGAATTCCAATTCACTGGCCGTCGTTT-3′. To generate pGEM-T7-sgNonsense_IVT (Supplementary Tables S1–S2), a fragment containing the T7-sgNonsense cassette was first created by PCR amplification of pUC19-U6-sgNonsense (Forward primer: 5′-GGAATTCTAATACGACTCACTATAGGGGGAGTTGTGTTTGTGGACGAAGGTT-3′; reverse primer: 5′-GCGGCCGCCTAATGGATCCTAGTACTCGAGAAAAAAAGCACCGACTCGGTGCCAC -3′). The PCR product was then cloned into a modified pGEM-11zf(+)-based vector, which was created by PCR amplification of pGEM-T7-sgCASFISH (Forward primer 5′- CTCGAGTACTAGGATCCATTAGGCGGCCGC-3′; reverse primer: 5′-CCCTATAGTGAGTCGTATTAGAATTCC-3′), using Gibson Assembly (Beijing Lablead Biotech Co., Ltd). pGEM-T7-sgNonsense-2xMS2_IVT and pGEM-T7-sgNonsense-MTS*α*_IVT (Supplementary Tables S1–S2) were also generated using Gibson Assembly, with the fragments containing T7-sgNonsense-2xMS2 cassette and T7-sgNonsense-MTS*α* cassette derived from pUC19-U6-sgNonsense-2XMS2 and pUC19-U6-sgNonsense-MTS*α*, respectively.

#### Other plasmids used in the study

To generate the *Streptococcus pyogenes* dCas9 expression plasmid pdCas9, the coding region of dCas9 was first PCR amplified from pSLQ1658-dCas9-EGFP, a gift from Dr. Bo Huang and Dr. Stanley Qi (Addgene plasmid #51023) [5], using forward primer 5′- ACTGCTGCTAGCGCTACCGGTCGCCACCATGGTGCCCAAAAAGAAGAGG-3′ and reverse primer 5′-ACCTGCGAATTCTTAGAACAGCTCCTCGCCC-3′. The PCR product was then inserted into the pEGFP-C1 vector (Clontech) digested with NheI and EcoRI to excise out EGFP. The MS2_EGFP and PP7_mCherry plasmids that encode MCP-EGFP and PCP-mCherry, respectively, were gifts form Dr. Daniel Larson (Addgene plasmids #61764 and #61763) [20]. To generate miRFP670-PSPC1 that encodes PSPC1 (also known as PSP1*α*) fused to miRFP670, the coding region of miRFP670 was first PCR amplified from pmiRFP670-N1, a gift from Dr. Vladislav Verkhusha (Addgene plasmid #79987) [21], using forward primer 5′-GGATCCACCGGTCGCCACCATG-3′ and reverse primer 5′-CTTGAGCTCGAGATCTGAGTCCGGAGCTCTCAAGCGCGGTGATCCGC-3′. The PCR product was then inserted into EYFP-PSP1*α*, a gift from Dr. Archa H. Fox (University of Western Australia, AUS) and Dr. Angus I. Lamond (University of Dundee, UK), digested with AgeI and XhoI to excise out EYFP. The bacterial expression plasmid pET302-6His-dCas9-Halo that encodes dCas9 fused to HaloTag (dCas9-HaloTag) was a gift from Dr. Wulan Deng and Dr. Robert Singer (Addgene plasmid #72269) [22].

### Synthesis of U6-sgRNA cassettes

Using the parental plasmids as templates, U6-sgRNA cassettes were obtained either by enzymatic digestion using EcoRI and HindIII followed by treatment with calf intestinal phosphatase (CIP) (New England Biolabs), or via PCR using forward primer 5′-AATTCGATCCGACGCGCCATCTCTA-3′ and reverse primer 5′-AGCTTAAAAAAAGCACCGACTCGGT-3′.

### Synthesis of molecular beacons (MBs)

The anti-MTS*α* MB was labeled with an Iowa Black FQ quencher at the 5′-end and an ATTO550 fluorophore at the 3′-end, and has the sequence: 5′-mCmUmCmAmG*mC*mG*mU*mA*mA*mG*mU*mG*mA*mU*mG*mU*mC*mG*mU*mG*mA* mCmUmGmAmG-3′ (Underlined letters indicate the MB stem; m represents 2′-*O*-methyl RNA modification; * represents phosphorothioate linkage modification). The anti-MTS*β* MB was labeled with an ATTO647N fluorophore at the 5′-end and an Iowa Black RQ quencher at the 3′-end, and has the sequence: 5′-mCmUmUmCmG*mU*mC*mC*mA*mC*mA*mA*mA*mC*mA*mC*mA*mA*mC*mU*mC*mC* mU*mGmAmAmG-3′. The MB sequences are designed to avoid hybridization with endogenous RNAs and the oligonucleotide modifications were designed to minimize nonspecific protein binding in mammalian cells [23]. All MBs were synthesized by Integrated DNA Technologies.

### Synthesis of fluorescently labeled dCas9 and *in vitro*-transcribed sgRNA

#### JF549-dCas9

dCas9-HaloTag was expressed and purified according to methods described previously [22], with some modifications. Briefly, the protein was expressed in *E. coli* Transetta (DE3) (TransGen Biotech) and was grown in LB medium at 16°C overnight following induction with 0.5 mM isopropyl β-D-1-thiogalactopyranoside (Inalco Pharmaceuticals). Cells were lysed by sonication in lysis buffer (20 mM HEPES, 500 mM NaCl, 0.5 mM TCEP, 20 mM imidazole, pH 7.5) supplemented with 1x protease inhibitor cocktail (Solarbio). Bacterial genomic DNA was precipitated by adding 0.1% (wt/vol) 70 kDa polyethylenimine (Shanghai Aladdin Biochemical Technology). Then the cell lysate was centrifuged at 21 100 x g for 30 min and the supernatant was filtered through a 0.45 μm filter, followed by precipitation of the protein with ammonium sulfate (Beijing Chemical Industrial Group). The precipitated protein was then collected by centrifugation at 10 000 x g for 20 min, and the resulting pellet was resuspended using lysis buffer followed by removal of large aggregates with a 0.45 μm syringe filter. The clarified lysate was then subjected to a 5-mL HisTrap HP column (Cytiva). The bound protein was then subjected to an imidazole step gradient elution (120 mM per step; final = 500 mM) in 20 mM HEPES, 500 mM NaCl, 0.5 mM TCEP, pH 7.5. The eluent was then buffer exchanged into 20 mM HEPES, 150 mM KCl, 0.5 mM TCEP pH 7.5 by a 50 000-MWCO centrifugal filter (Millipore Amicon) prior to loading onto a 5-mL HiTrap SP HP column (Cytiva). This was followed by a KCl linear gradient elusion (final = 1 M) in 20 mM HEPES, 0.5 mM TCEP, pH 7.5 to recover the bound protein. The purified protein was then buffer exchanged into 20 mM HEPES, 150 mM KCl, 0.5 mM TCEP, pH 7.5 using dialysis (Thermo Scientific). To synthesize JF549-dCas9, the purified dCas9-HaloTag was mixed with Janelia Fluor^®^ 549 HaloTag^®^ Ligand (Promega) at a 1:8 molar ratio and incubated at room temperature (RT) for 30 min, followed by incubation at 4°C overnight. The JF549-dCas9 was purified twice using Zeba^TM^ spin desalting columns (40K MWCO, 0.5 mL) (Thermo Fisher) in 50 mM HEPES (pH 7.5), 150 mM KCl, 1 mM TCEP, 10% (vol/vol) glycerol.

#### In vitro-transcribed sgRNA

pGEM-T7-sgCASFISH, pGEM-T7-sgNonsense_IVT, pGEM-T7-sgNonsense-2xMS2_IVT and pGEM-T7-sgNonsense-MTS*α*_IVT were linearized with XhoI and then used to transcribe sgCASFISH, sgNonsense_IVT, sgNonsense-2xMS2_IVT and sgNonsense-MTS*α*_IVT, respectively, using RiboMAX™ Large-Scale RNA Production System-T7 (Promega). The transcripts were purified using Monarch^®^ RNA Cleanup Kit (50 μg) (New England Biolabs) and dissolved in nuclease-free water (Ambion).

### Cell culture

hTERT RPE-1 (RPE-1) cells (American Type Culture Collection) were cultured in Dulbecco's Modified Eagle Medium/Nutrient Mixture F-12 (DMEM/F12, Gibco), supplemented with 10% (vol/vol) FBS (Gemini Bio) and 0.01 mg/mL Hygromycin B (Cellgro). HEK293, HeLa cells, and primary BJ fibroblast (BJ) cells (American Type Culture Collection) were cultured in DMEM (Corning), supplemented with 10% (vol/vol) FBS and 1x GlutaMAX^TM^ (Thermo Fisher). All cells were cultured at 37°C, 5% (vol/vol) CO_2_, and 90% relative humidity. All experiments were performed with cells at passage numbers between 5 and 25.

### Transfection of plasmids and U6-sgRNA cassettes

Nucleofection was performed using Neon^®^ Transfection System. Specifically, cells grown to 70% confluency were trypsinized, washed with 1x PBS and pelleted, followed by resuspension in 11 μL of 1x PBS containing indicated amounts (see Figure legends) of plasmids or U6-sgRNA cassettes to obtain final cell concentration of 5000 cells per μL. Thereafter, 10 μL of the cell mixture were nucleofected with the parameters set at 1050 V with a 30 ms pulse width and two pulses total for RPE-1 cells, 1150 V with a 20 ms pulse width and two pulses total for HEK293 cells, 1005 V with a 35 ms pulse width and 2 pulses total for HeLa cells, and 1650 V with a 20 ms pulse width and 1 pulse total for BJ cells. Following nucleofection and two washes in culture medium, the cells were seeded on 8-well Lab-Tek^TM^ chambered coverglass previously coated with 0.01% poly-L-lysine (Sigma-Aldrich) (for 1,6-hexanediol treatment) or 10 μg/mL fibronectin (Sigma-Aldrich) (for other experiments).

FuGENE^®^ 6 (Promega) and Lipofectamine^TM^ 2000 (Life Technologies)-based transfections were performed according to the manufacturers’ protocols. Specifically, cells grown to 50–70% confluency on 8-well Lab-Tek^TM^ chambered coverglass were transfected with 250 ng (i.e. 0.13 pmol) of pUC19-U6-sgNonsense.

### Treatments with DNase I, RNase A, actinomycin D, 1,6-hexanediol, and 2,5-hexanediol

#### DNase I

Cells were fixed and permeabilized with a prechilled solution of methanol and acetic acid at a 1:1 volume ratio at -20°C for 20 min, followed by three washes with 1x PBS. After a 5-min incubation in 0.2 M HCl containing 0.02% (wt/vol) pepsin (VWR Life Science) at 37°C and two washes with 1x PBS, the cells were incubated with 20 U/mL DNase I (New England Biolabs) in DNase I buffer at 37°C for 2 h and then washed twice with 1x PBS.

#### RNase A

Cells were fixed with 1% (vol/vol) formaldehyde (J&K Scientific) in 1x PBS at RT for 10 min, washed twice with 1x PBS, and then permeabilized with 0.5% (vol/vol) Triton-X-100 (Sigma-Aldrich) in 1x PBS at RT for 10 min. After two washes with 1x PBS, the cells were incubated with 0.1 mg/mL RNase A (Monarch) in 1x PBS at 37°C for 30 min and then washed twice with 1x PBS.

#### Actinomycin D

Cells at 18 h post-nucleofection were incubated with 10 μg/mL actinomycin D (MedChemExpress) in culture medium at 37°C for 6 h before fixation.

#### 1,6-hexanediol

Cells were incubated with 8% (wt/vol) 1,6-hexanediol (Aladdin) in culture medium at 24 h post-nucleofection at 37°C for 5 min before fixation.

#### 2,5-hexanediol

Cells were incubated with 8% (wt/vol) 2,5-hexanediol (Aladdin) in culture medium at 24 h post-nucleofection at 37°C for 5 min before fixation.

### RNA fluorescence *in situ* hybridization (FISH)

#### Synthesis of RNA FISH probes

RNA FISH probes complementary to nonsense sequences (Supplementary Table S4) were synthesized by Integrated DNA Technologies. RNA FISH probes complementary to cryptic plasmid transcripts (CPTs) were synthesized by reacting ATTO647N NHS ester (Sigma-Aldrich) or Alexa Fluor^TM^ 488 carboxylic acid, succinimidyl ester (Thermo Fisher) with NH_2_-modified oligonucleotides (Supplementary Table S4) (synthesized by Sangon biotech) at a dye to oligonucleotide molar ratio of 5:1 in 1xPBS at RT overnight. The fluorescent conjugates were purified on PD-10 desalting columns (Cytiva) gel in 1x PBS. Successful labeling was confirmed spectrophotometrically. anti-CPT_1 was designed to be complementary to the origin of replication (*ori*) commonly found in plasmid vectors, which can strongly initiate the transcription of cryptic transcripts [17, 19]. anti-CPT_2 was designed to be complementary to a sequence that is located 49-bp downstream of the U6-sgRNA cassette. The sequence of anti-CPT_2 was chosen more randomly, based on the deep sequencing results suggesting that the entire plasmid backbone may be subjected to cryptic transcription [18].

#### RNA FISH experiments

Cells were fixed with 1% (vol/vol) formaldehyde in 1x PBS at RT for 10 min, washed twice with 1x PBS, and then permeabilized with 0.5% (vol/vol) Triton-X-100 in 1x PBS at RT for 10 min. After two washes with 1x PBS and then two 5-min incubations in RNA FISH wash buffer (2x saline-sodium citrate (SSC) (Life Technologies), 10% (vol/vol) formamide (Ambion)), the cells were incubated in RNA FISH hybridization buffer (10% (vol/vol) dextran sulfate (VWR Life Science), 2x SSC, 10% (vol/vol) formamide) containing FISH probes (25 nM for ATTO647N-tagged probes and 250 nM for Alexa Fluor^TM^ 488-tagged probes) in a humidified chamber at 37°C overnight. On the following day, the cells were washed twice with wash buffer and then incubated in wash buffer at 37°C for 30 min, followed by two washes with 2x SSC and one wash with 1x PBS to remove unhybridized probes. For experiments where NEAT1_2 (the longer isoform of NEAT1) was also labeled, the samples were further incubated in RNA FISH hybridization buffer containing 25 nM of a pool of Quasar^®^ 570-labeled, human NEAT1_2 Stellaris RNA FISH probes (SMF-2037–1, Biosearch Technologies) that specifically target the middle region of NEAT1_2 in a humidified chamber at 37°C overnight and then washed as described above.

### Cas9-mediated DNA FISH (CASFISH)

CASFISH was performed according to methods described previously [22], with some modifications. In brief, cells were fixed and permeabilized with a pre-chilled solution of methanol and acetic acid at a 1:1 volume ratio at −20°C for 20 min, followed by three washes with 1x PBS. Thereafter, the cells were incubated in blocking/reaction buffer (20 mM HEPES (pH 7.5), 100 mM KCl, 5 mM MgCl_2_, 5% (vol/vol) glycerol, and 0.1% (vol/vol) TWEEN-20) at 37°C for 15 min. To assemble JF549-dCas9 and sgCASFISH, the purified sgCASFISH was first heated to 65°C for 5 min and then slowly cooled to RT. 250 nM of JF549-dCas9 was then mixed with sgCASFISH at a 1:4 molar ratio in 1x PBS and incubated at RT for 10 min. The pre-blocked cells were then applied with 5 nM of the protein-RNA mixture in fresh blocking/reaction buffer and then incubated at 37°C for 30 min. Unbound probes were removed by three washes with 1x PBS.

### Combined CASFISH and RNA FISH

Following CASFISH as described above, the cells were post-fixed with 4% (vol/vol) paraformaldehyde (Electron Microscopy Sciences) in 1x PBS at RT for 30 min, washed twice with 1x PBS, and then incubated with 70% (vol/vol) ethanol at 4°C overnight. On the following day, the samples were washed twice in RNA FISH wash buffer and then incubated with RNA FISH probes as described above.

### Combined CASFISH and immunofluorescence

Following CASFISH as described above, the cells were post-fixed with 1% (vol/vol) formaldehyde in 1x PBS at RT for 10 min, washed twice with 1x PBS, and then incubated with 0.5% (vol/vol) Triton-X-100 at RT for 10 min. After two washes with 1x PBS, the cells were incubated with primary antibodies against TRIM19/PML diluted in 1x PBS containing 2% (vol/vol) FBS at RT for 30 min (see Supplementary Table S5 for antibody usage), followed by three washes with 1x PBS. The cells were then incubated with Alexa Fluor^TM^ 488-conjugated secondary antibodies (Life Technologies) diluted in 1x PBS at RT for 1 h, followed by three washes with 1x PBS.

### Combined RNA FISH and immunofluorescence

Following RNA FISH described above, the cells were incubated with primary antibodies diluted in corresponding antibody dilution buffer (see Supplementary Table S5 for antibody usage), followed by three washes with 1x PBS. The cells were then incubated with Alexa Fluor^TM^ 488-conjugated secondary antibodies diluted in 1x PBS at RT for 1 h, followed by three washes with 1x PBS.

### RNAi transfection and western blot

FUS siRNA (siFUS: 5′-CGGACAUGGCCUCAAACGATT-3′), SFPQ siRNA (siSFPQ: 5′-GGAAGAUGCCUAUCAUGAATT-3′) and control siRNA (siCtrl: 5′-UUCUCCGAACGUGUCACGUTT-3′) were purchased from GenePharma. RPE-1 cells seeded in a 24-well tissue culture plate were transfected with 10 pmol of the indicated siRNAs using Lipofectamine^TM^ RNAiMax (Life Technologies), according to the manufacturer's instructions. At 48 h post-transfection, cells were trypsinized and then nucleofected with the pUC19-U6-sgNonsense plasmid as described above, followed by seeding onto an 8-well Lab-Tek^TM^ chambered coverglass or another 24-well tissue culture plate. At 24-h post-nucleofection, the cells cultured in the chambered coverglass were subjected to RNA FISH experiments as described above and those in the 24-well tissue culture plate were subjected to cell lysis for western blot analysis with antibodies against FUS, SFPQ and GAPDH (as loading control). See Supplementary Table S5 for antibody usage.

### MS2/PP7 dual-color live-cell imaging

To visualize plasmid-expressed locus-targeting sgRNAs, cells were nucleofected with 865 ng (i.e. 0.45 pmol) of pUC19-U6-sgChr3q29_1–2XMS2 plus pUC19-U6-sgChr3q29_2–2XPP7 (prepared at a 1:1 molar ratio), together with 150 ng of pdCas9 and 20 ng each of MS2_EGFP and PP7_mCherry. To visualize cassette-expressed locus-targeting sgRNAs, cells were nucleofected with 134 ng (i.e. 0.45 pmol) of U6-sgChr3q29_1–2XMS2 plus U6-sgChr3q29_2–2XPP7 cassettes (prepared at a 1:1 molar ratio), together with the indicated amounts of pdCas9, MS2_EGFP, and PP7_mCherry. In experiments where PSPC1 was also visualized, 20 ng of miRFP670-PSPC1 was also nucleofected. Thereafter, the cells were incubated for 24–48 h prior to imaging.

### CRISPR/MB dual-color live-cell imaging

To visualize plasmid-expressed locus-targeting sgRNAs, cells were nucleofected with 870 ng (i.e. 0.45 pmol) of pUC19-U6-sgChr3q29_1-MTS*β* plus pUC19-U6-sgChr3q29_2-MTS*α* (prepared at a 1:1 molar ratio), together with 150 ng of pdCas9. To visualize cassette-expressed locus-targeting sgRNAs, cells were nucleofected with 139 ng (i.e. 0.45 pmol) of U6-sgChr3q29_1-MTS*β* plus U6-sgChr3q29_2-MTS*α* cassettes (prepared at a 1:1 molar ratio), together with 150 ng of pdCas9. At 10 h post-nucleofection, the cells were nucleofected with 0.5 μM each of anti-MTS*β* MB and anti-MTS*α* MB according to methods described previously [23–25]. Thereafter, the cells were incubated for 24 h prior to imaging.

### Fluorescence microscopy

Fluorescence microscopy experiments were performed on an Olympus IX 83 motorized inverted fluorescence microscope equipped with a back-illuminated EMCCD camera (Andor), a 40x UPlanSApo 0.95NA or a 100x UPlanSApo 1.4NA objective lens, and Sutter excitation and emission filter wheels under the control of the cellSens Dimension software. Co-labeling experiments with ATTO647N and JF549 or Quasar^®^ 570-based probes were performed using IX3-U-m4TIRSbx and a DV2-cube (ET585/65m, 635lpxr, ET655lp, Photometrics), with the excitation light of ATTO647N provided by a 640 nm laser line (140 mW) and the excitation light of JF549/Quasar^®^ 570 provided by a 561 nm laser line (150 mW). For other experiments, images of DAPI, EGFP/Alexa Fluor^TM^ 488 and ATTO550/JF549 were acquired using the Olympus MT20 filter set for DAPI, EGFP and TAMRA, images of mCherry were acquired using a Chroma filter set (ET560/40x, T585lpxr, ET630/75m), and images of ATTO647N/miRFP670 were acquired using a Chroma filter set (ET620/60x, ET700/75m, T660lpxr), with the excitation light provided by a X-Cite Series 120 light source housing a Mercury Lamp (EXFO). Three-dimensional (3D) image stacks were acquired with 0.25 μm increments in the z-direction. All images were analyzed using Fiji [26] and the AutoQuant deconvolution software (MediaCybernetics).

### Quantifying nonspecific sgRNA foci detected by FISH

For each cell, a 3D image stack was acquired to generate a maximum intensity projection image. Then, signal-to-background ratio (SBR) was calculated for each candidate spot, as described previously [7], according to the following formula:


\begin{equation*}SBR = \frac{{{{F}_{candidate\ spot}}}}{{{{F}_{nucleus}}}}\end{equation*}

where *F_candidate spot_* refers to the maximum spot fluorescence intensity, and *F_nucleus_* refers to the average intensity of a region of interest (ROI) drawn in the nucleoplasm outside of spots. The maximum SBR value of 1.60 determined in mock nucleofected cells was used as the threshold for defining nonspecific RNA foci.

### Analysis of % cells with colocalized spot(s) and % cells with uncolocalized spot(s) for dual-color live-cell imaging

3D image stacks were acquired for each effector probe fluorescence channel for each cell. Following the determination of colocalized spots and uncolocalized spots (by visual examination), % cells with colocalized spot(s) and % cells with uncolocalized spot(s) were calculated by dividing the number of cells containing colocalized spots and uncolocalized spots, respectively, by the total number of cells containing both effector probe signals (spots).

### Calculation of Pearson's correlation coefficient

For each cell, two 3D image stacks, one corresponding to a different fluorescence cannel, were acquired. Following generation of the maximum intensity projection images for the stacks, equal-sized ROIs were applied to obtain a pair of cropped images containing the nucleus. Pearson's correlation coefficient was calculated for the cropped images using Fiji's “Colocalization Finder” plugin.

### Statistical analysis

All statistical analyses were performed using either two-tailed Student's t-test or one-way analysis of variance (ANOVA) with post hoc testing of pairwise comparisons by Dunnett's T3 test using IBM SPSS Statistics, version 27.

## Results

### False-positive foci formation is a plasmid-dependent process, regardless of sgRNA scaffold design

We generated a pUC19-based and U6-promoter-driven plasmid encoding sgRNA harboring the original sgRNA scaffold [1] and a nonsense spacer sequence that does not have any known endogenous targets, so that any incidence of foci formation may be considered a nonspecific activity, and this allowed us to better isolate and characterize the issue of false-positive foci formation without interference from authentic genomic loci labeling. The sgRNA and its producer plasmid are denoted as sgNonsense and pUC19-U6-sgNonsense, respectively. Following nucleofection of the plasmid into hTERT RPE-1 (RPE-1) cells, RNA fluorescence *in situ* hybridization (FISH) experiments were performed with an oligonucleotide probe whose sequence is complementary to the nonsense spacer, termed the anti-Nonsense probe (see Materials and Methods).

**Figure 1.**
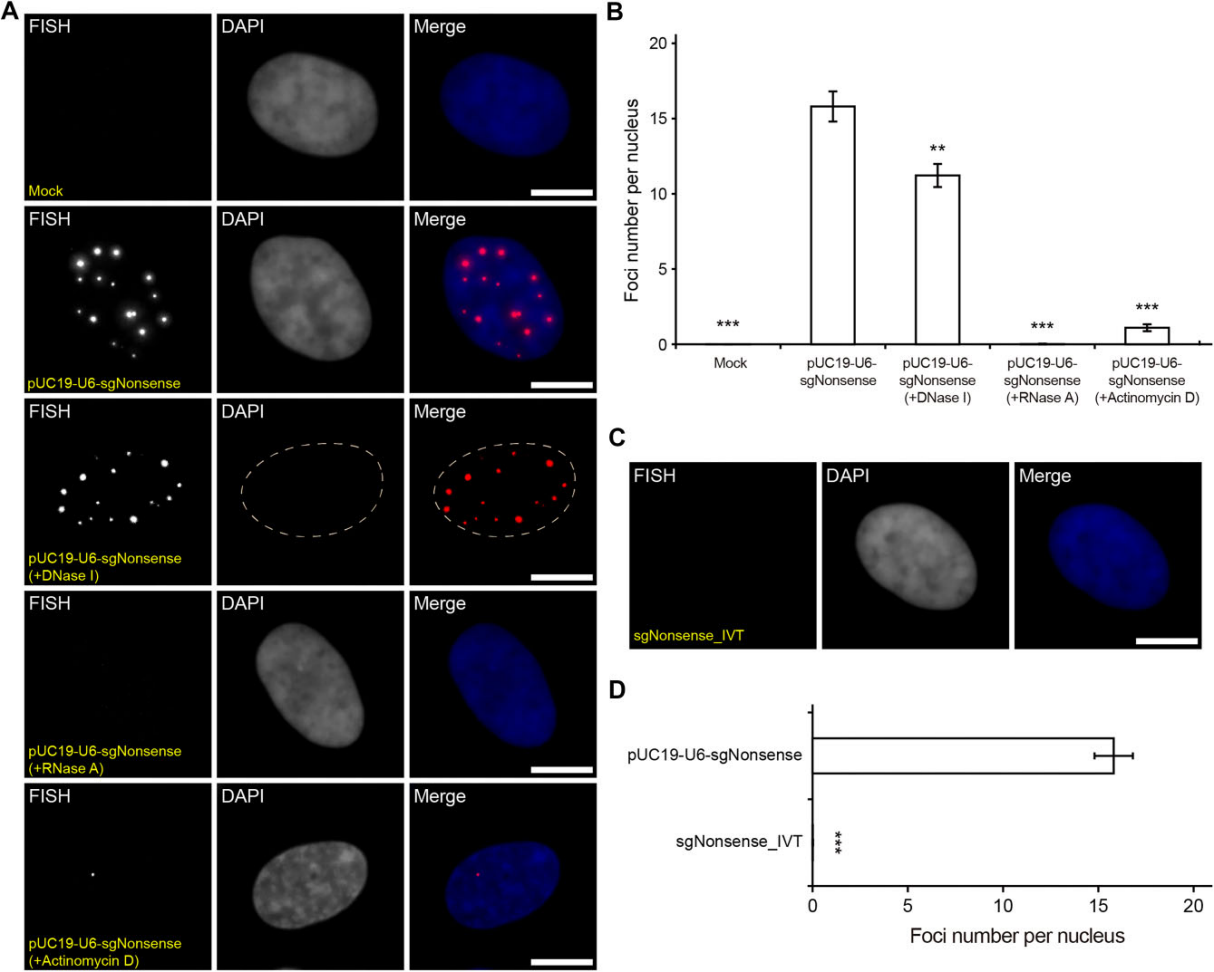
Transfecting sgRNA-expression plasmids, but not sgRNAs, results in false-positive foci formation. (**A**-**B**) RPE-1 cells were mock nucleofected or nucleofected with 0.45 pmol of pUC19-U6-sgNonsense. pUC19-U6-sgNonsense-transfected cells were further treated with DNase I, RNase A or actinomycin D and then subjected to RNA FISH with the anti-Nonsense probe. Note that cell fixation and permeabilization was performed before the DNase I and RNase A treatments and after the actinomycin D treatment. (**A**) Representative maximum intensity projection images. The nucleus is indicated either by DAPI staining or dashed line. (**B**) Average numbers of FISH foci detected in cells from (**A**). N = 63, 116, 106, 98, and 60 cells for the mock, pUC19-U6-sgNonsense, pUC19-U6-sgNonsense (+DNase I), pUC19-U6-sgNonsense (+RNase A), and pUC19-U6-sgNonsense (+Actinomycin D) samples, respectively. (**C-D**) RPE-1 cells were nucleofected with 4 μM of the *in vitro*-transcribed sgRNA, sgNonsense_IVT. At 24 h post-nucleofection, the cells were subjected to RNA FISH with the anti-Nonsense probe. (**C**) A representative maximum intensity projection image. DAPI stains the nucleus. (**D**) The average number of FISH foci detected in sgNonsense_IVT-transfected cells from (**C**) (*n* = 68 cells), plotted together with data of pUC19-U6-sgNonsense-transfected cells from (**B**) (*n* = 116 cells). All data represent mean ± SEM. Asterisks indicate significant differences from (untreated) pUC19-U6-sgNonsense-transfected cells (** *P* < 0.01, *** *P* < 0.001). Scale bar, 10 μm.

Fluorescence microscopy imaging showed that, at 24 h post-nucleofection, discrete foci were readily observed in the nucleus of pUC19-U6-sgNonsense-transfected cells (15.80 ± 1.01 foci per cell) but not of mock-transfected cells (Fig. 1A and B). The observed foci came from probe hybridization to the nonsense RNA sequence as opposed to its DNA template, since they were readily eliminated by treatment with RNase A or a global transcription inhibitor actinomycin D, but not with DNase I (Fig. 1A and B). Nonspecific foci were also observed when the plasmid was transfected into different cell types (HEK293, HeLa, and primary BJ fibroblast cells) (Supplementary Fig. S1A), when other transfection methods were used (Supplementary Fig. S1B), or when cells were transfected with different plasmids containing a different promoter (CMV), a different vector (pGEM-11zf(+)), or a different nonsense sgRNA (Supplementary Fig. S1C-E). Furthermore, modified sgNonsense scaffolds incorporating the MS2 aptamer [27] or a molecular beacon (MB) target sequence [28] could also form nonspecific foci as assessed by FISH (Supplementary Fig. S2A and C) or live-cell imaging (Supplementary Movie S1). Notably, cells nucleofected with *in vitro*-transcribed (IVT) unmodified or modified sgNonsense exhibited little if any capacity to form nonspecific foci as assessed by FISH (Fig. 1C and D and Supplementary Fig. S2B and C). These findings, together with previous studies showing fluorescently tagged, synthetic gRNAs do not form nonspecific foci [7], have collectively indicated that the false-positive foci formation is a plasmid-dependent process, regardless of sgRNA scaffold design.

### Cryptic transcripts derived from plasmid backbones can contribute to false-positive foci formation

**Figure 2.**
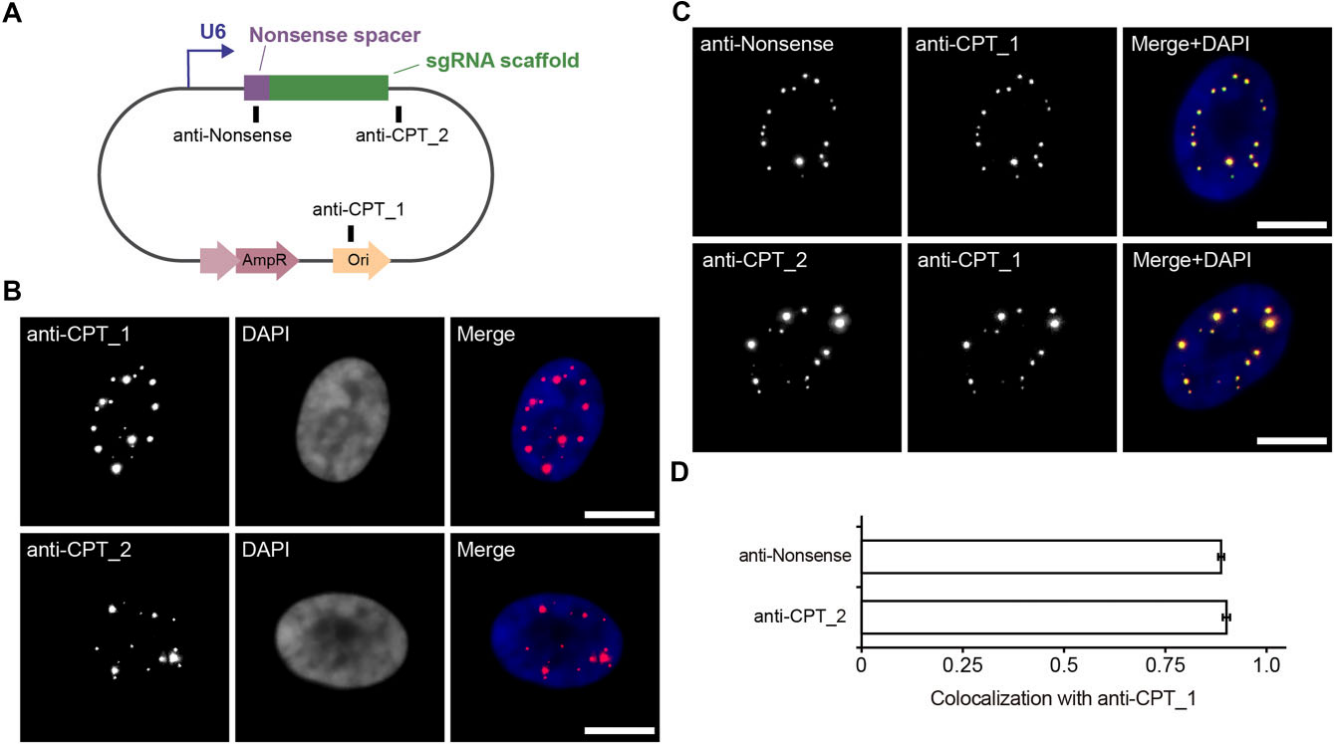
Cryptic transcripts from the sgRNA-expression plasmid pUC19-U6-sgNonsense contribute to false-positive foci formation. (**A**) Schematic diagram showing the positions of the RNA FISH (anti-Nonsense, anti-CPT_1, and anti-CPT_2) target sequences within pUC19-U6-sgNonsense. (**B**) RNA foci were detected by anti-cryptic plasmid transcript (CPT) probes, namely anti-CPT_1 and anti-CPT_2 probes. RPE-1 cells were nucleofected with 0.45 pmol of pUC19-U6-sgNonsense. At 24 h post-nucleofection, the cells were subjected to RNA FISH using ATTO647N-conjugated anti-CPT_1 or anti-CPT_2 probes. Representative maximum intensity projection images are shown. (**C** and **D**) Colocalization between RNA FISH foci detected by the anti-CPT_1 probe and those detected by the anti-Nonsense or the anti-CPT_2 probes. RPE-1 cells were nucleofected with 0.45 pmol of pUC19-U6-sgNonsense. At 24 h post-nucleofection, cells were subjected to co-labeling RNA FISH experiments using the anti-CPT_1 probe (Alexa Fluor^TM^ 488-conjugated) with the anti-Nonsense probe (ATTO647N-conjugated), or the anti-CPT_2 probe (ATTO647N-conjugated). (**C**) Representative maximum intensity projection images. (**D**) Colocalization (Pearson's correlation coefficients) between anti-CPT_1 signals and anti-Nonsense (*n* = 49 cells) or anti-CPT_2 signals (*n* = 36 cells) in cells from (**C**). Data represent mean ± SEM. DAPI stains the nucleus. Scale bar, 10 μm.

The observation that transfecting nonsense sgRNA expression plasmids, but not nonsense IVT sgRNAs, results in RNA foci suggests that plasmids are generating additional RNAs containing sequences that can be bound by fluorescent probes. Such RNAs, denoted as cryptic plasmid transcripts, arise from spurious transcriptional activities that may initiate within backbone regions of many plasmids [16–19, 29, 30] and can vary greatly in length and quantity [17, 18]. Thus, cryptic plasmid transcripts can harbor portions of sgRNAs (either full-length or partial) that can be fluorescently tagged. To test if cryptic plasmid transcripts contributed to false-positive foci formation, we performed RNA FISH experiments in pUC19-U6-sgNonsense-transfected cells using two additional probes, termed the anti-cryptic plasmid transcript (CPT)_1 and anti-CPT_2 probes (see “Materials and methods” for probe design rationales), whose sequences are complementary to plasmid backbone regions that are distal and proximal to the U6-sgRNA cassette, respectively (Fig. 2A and Supplementary Table S4).

Notably, each anti-CPT probe could exhibit a punctate fluorescence staining pattern indicative of RNA foci formation (Fig. 2B and Supplementary Fig. S3), similar to that observed with the anti-Nonsense probes (Fig. 1A and Supplementary Figs S1 and S2A). In addition, dual-color imaging using the anti-CPT_1 probe (Alexa Fluor^TM^ 488-conjugated) in conjunction with either the anti-Nonsense probe (ATTO647N-conjugated) or the anti-CPT_2 probe (ATTO647N-conjugated) showed strong colocalizations (Pearson's correlation coefficients ∼ 0.90) between the Alexa Fluor^TM^ 488-positive and the ATTO647N-positive foci (Fig. 2C and D). Thus, despite differences in the probes, both anti-CPT probes and the anti-Nonsense probe labeled the same RNA foci. Furthermore, additional experiments with pGEM and pcDNA3.1-based vectors demonstrated that cryptic transcripts derived from these plasmids could also nonspecifically accumulate to form false-positive foci when detected using the anti-CPT_1 and anti-CPT_2 probes (Supplementary Fig. S4). Based on these findings and the data showing the absence of IVT sgRNA foci in cells (Fig. 1C and D and Supplementary Fig. S2B and C), it appears that false-positive foci arise from cryptic plasmid transcripts rather than the intended sgRNAs.

### Identifying molecular players responsible for false-positive foci formation

**Figure 3.**
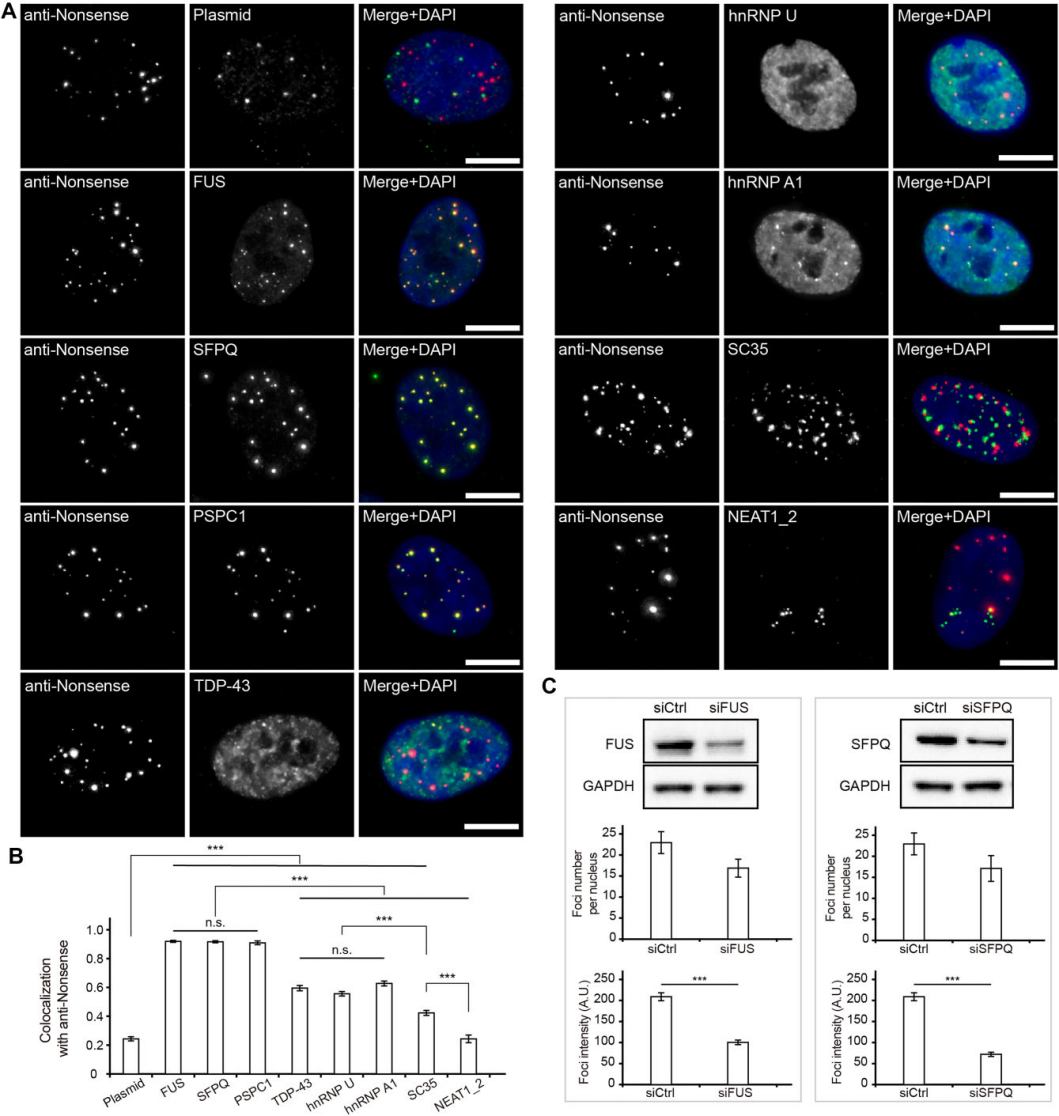
Cryptic RNA foci require paraspeckle proteins to form. (**A**-**B**) Colocalizations between anti-Nonsense-positive foci and plasmid DNA, RNA binding proteins or NEAT1_2 RNA. RPE-1 cells were nucleofected with 0.45 pmol of pUC19-U6-sgNonsense. At 24 h post-nucleofection, cells were subjected to different combinations of RNA FISH, CASFISH and immunofluorescence using antibodies against the indicated proteins (see Materials and Methods). (**A**) Representative maximum intensity projection images. DAPI stains the nucleus. Scale bar, 10 μm. (**B**) Colocalization (Pearson's correlation coefficients) between anti-Nonsense signals and fluorescence signals of the indicated molecules. N = 50, 36, 34, 37, 41, 40, 56, 37, and 31 cells for colocalizations with plasmid, FUS, SFPQ, PSPC1, TDP-43, hnRNP U, hnRNP A1, SC35, and NEAT1_2, respectively. n.s., not significant. (**C**) The effect of knockdown of FUS or SFPQ on false-positive foci formation. FUS siRNA (siFUS), SFPQ siRNA (siSFPQ) or negative control siRNA (siCtrl) were transfected into RPE-1 cells. At 48-h post-transfection, the cells were nucleofected with 0.45 pmol of pUC19-U6-sgNonsense, followed by seeding onto a 24-well tissue culture plate or an eight-well Lab-Tek^TM^ chambered coverglass. At 24 h post-nucleofection, knockdown efficiencies and false-positive foci formation were assessed by western blot and RNA FISH (using anti-Nonsense), respectively (see “Materials and methods”). (*Top panels*) siFUS treatment reduced the FUS level by ∼76% and siSFPQ reduced the SFPQ level by ∼67%. (*Middle panels*) Average numbers of FISH foci detected in cells treated with different siRNAs. *N* = 30 cells for each sample. (*Bottom panels*) Average intensities of FISH foci detected in cells treated with different siRNAs. *N* = 441 foci from 30 siCtrl-treated cells, 430 foci from 30 siFUS-treated cells, and 389 foci from 30 siSFPQ-treated cells. A.U., arbitrary unit. All data represent mean ± SEM. Asterisks indicate significant differences (*** *P* < 0.001).

Given a single fluorophore is too dim to be distinguishable from background in the cellular environment [31, 32], we interpreted that the observed foci result from nonspecific accumulation of multiple cryptic plasmid transcripts. To clarify the nature of this accumulation, we first examined whether the cryptic plasmid transcripts and their producer plasmids share the same spatial distributions in cells transfected with pUC19-U6-sgNonsense. The cryptic plasmid transcripts were visualized by RNA FISH using the anti-Nonsense probe and their producer plasmids were visualized by Cas9-mediated DNA-FISH (CASFISH) [22] with the spacer sequence designed to target an sgRNA scaffold encoding plasmid region. The majority of the cryptic transcript foci and their producer plasmid foci were found to be fairly separated in space (>500 nm) and exhibited poor colocalization (Pearson's correlation coefficient ∼0.24) (Fig. 3A and B). Further studies with immunofluorescence showed strong colocalization between the promyelocytic leukemia (PML) nuclear body protein TRIM19/PML and the plasmid DNA but not RNA (Supplementary Fig. S5), consistent with previous reports indicating foreign DNAs but not RNA transcripts have the tendency to accumulate at or near the peripheries of PML bodies [33–35], which presumably facilitate plasmid transcription [36]. Thus, following transcription, cryptic plasmid transcripts appeared to be quickly transported and sequestered elsewhere rather than accumulating around their producer plasmids.

**Figure 4.**
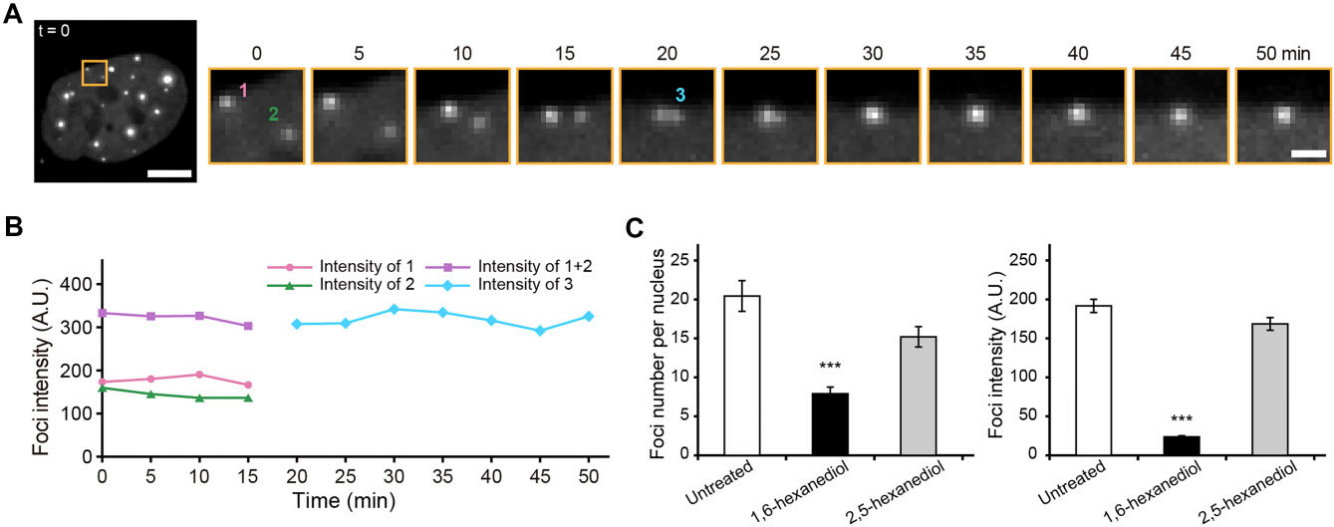
Cryptic RNA foci display liquid-like properties. (**A**-**B**) A cryptic RNA foci fusion event. RPE-1 cells were nucleofected with 20 ng of MS2_EGFP and 867 ng (i.e. 0.45 pmol) of pUC19-U6-sgNonsense-2XMS2, and imaged at 24-h post-nucleofection. (**A**) (*Left panel*) Maximum intensity projection image of a live cell containing a fusion event. Scale bar, 5 μm. (*Right panel*) Time lapse images of a fusion event between the two spots in the boxed region in the left panel. The two spots are denoted as 1 and 2. Note that at the 20 min time point, they appear to merge to form a new spot (denoted as 3). Images have been corrected for photobleaching. Scale bar, 1 μm. (**B**) Combined and individual foci fluorescence intensities of the labeled spots from (**A**) as a function of time. A.U., arbitrary unit. (**C**) Effects of 1,6-hexanediol and 2,5-hexanediol on cryptic RNA foci formation. RPE-1 cells were nucleofected with 0.45 pmol of pUC19-U6-sgNonsense. At 24 h post-nucleofection, the cells were treated with 8% (wt/vol) of either 1,6-hexanediol or 2,5-hexanediol for 5 min, and then subjected to RNA FISH using anti-Nonsense. (*Left panel*) Average numbers of anti-Nonsense-positive foci. *N* = 30 cells for each sample. (*Right panel*) Fluorescence intensities of individual foci. A.U., arbitrary unit. *N* = 276 foci from 30 untreated cells, 195 foci from 30 1,6-hexanediol-treated cells, and 194 foci from 30 2,5-hexanediol-treated cells. Data represent mean ± SEM for (**C**). Asterisks indicate significant differences from the untreated sample (*** *P* < 0.001).

Many, if not all, RNAs exist and function in association with RNA-binding proteins inside cells [37–39]. This led us to speculate that certain RNA-binding proteins were responsible for the rapid disengagement of cryptic plasmid transcripts from the producer plasmids. To test this, we performed RNA FISH to locate cryptic plasmid transcripts and immunofluorescence to survey their potential binding protein partners in pUC19-U6-sgNonsense-transfected cells. These proteins are FUS, SFPQ, PSPC1, TDP-43, hnRNP U, hnRNP A1, and SC35, which are RNA binding proteins and have been observed to form nuclear bodies [40–44]. Notably, cryptic plasmid transcripts showed more extensive colocalizations with FUS, SFPQ and PSPC1 (Pearson's correlation coefficients > 0.90) compared to the other tested proteins (Fig. 3A and B). Additionally, cryptic plasmid transcripts derived from modified sgRNA-expression plasmids, tagged by MCP-EGFP, also colocalized more readily with FUS, SFPQ, and PSPC1 than the other nuclear markers tested (Supplementary Fig. S6). Partial knockdown of FUS or SFPQ led to significant reduction in cryptic plasmid transcripts foci formation (Fig. 3C), confirming a regulation role of these proteins in mediating the disengagement of cryptic plasmid transcripts from plasmids. Furthermore, this regulation potentially involves liquid-liquid phase separation (LLPS), since the cryptic RNA foci displayed biophysical properties reminiscent of liquid droplets including sphericality, fusion competence (Fig. 4A and B), and sensitivity to 1,6-hexanediol (Fig. 4C), and FUS, SFPQ, and PSPC1 have been reported to drive LLPS [45–50]. Finally, cryptic plasmid transcripts do not colocalize with the long noncoding RNA nuclear paraspeckle assembly transcript 1 (i.e. NEAT1_2) (assessed by dual-color RNA FISH, see “Materials and methods”) (Fig. 3A and B), which normally serves as a scaffold to bind FUS, SFPQ and PSPC1 and mediate the formation of paraspeckles [40]. Together, these findings suggest that following transcription, cryptic plasmid transcripts are quickly recognized and shuttled away from their producer plasmids by FUS, SFPQ, and PSPC1 to form nuclear bodies that exhibit liquid-like properties.

### Transfecting U6-sgRNA cassettes results in marginal false-positive foci formation

**Figure 5.**
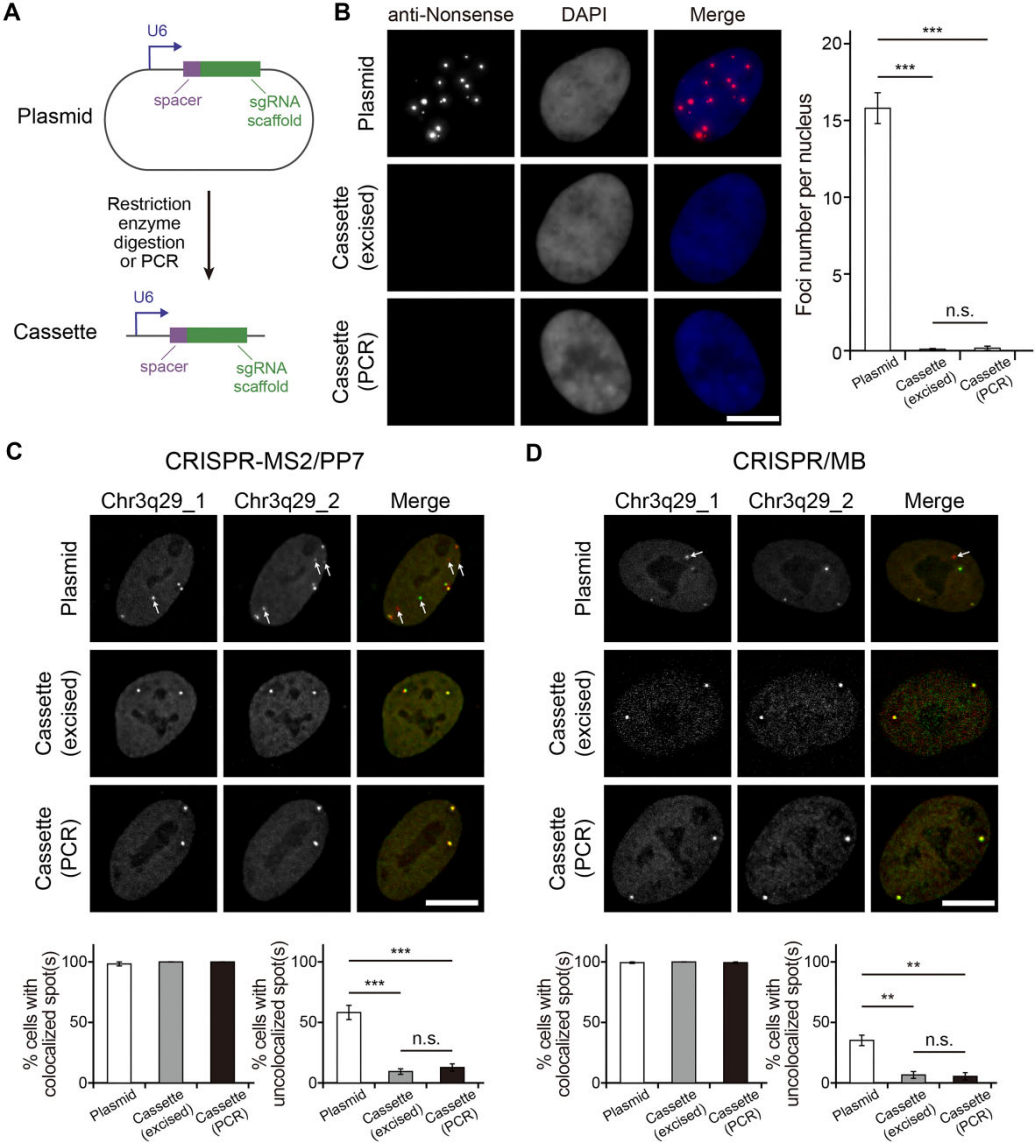
sgRNAs expressed from U6-sgRNA cassettes elicit marginal false-positive foci formation and confer more accurate genomic imaging in living cells. (**A**) Schematics of the plasmid and the U6-sgRNA cassette used for sgRNA expression. (**B**) Assessment of false-positive foci formation. RPE-1 cells were nucleofected with 0.45 pmol of pUC19-U6-sgNonsense, 0.45 pmol of the excised U6-sgNonsense cassette or 0.45 pmol of the PCR-derived U6-sgNonsense cassette. At 24 h post-nucleofection, the cells were subjected to RNA FISH using the anti-Nonsense probe. (*Left panel*) Representative maximum intensity projection images are shown. DAPI stains the nucleus. (*Right panel*) The average number of FISH foci detected in cells nucleofected with 0.45 pmol of the excised U6-sgNonsense cassette (*n* = 75 cells) and in cells nucleofected with 0.45 pmol of the PCR-derived U6-sgNonsense cassette (*n* = 31 cells), plotted together with the data of cells nucleofected with 0.45 pmol of pUC19-U6-sgNonsense from Fig. 1B (*n* = 116 cells). (**C** and **D**) Plasmids encoding sgRNAs with modified scaffolds and spacer sequences designed to target two different repetitive loci within the same genomic region Chr3q29, denoted as Chr3q29_1 and Chr3q29_2, were constructed for both (**C**) CRISPR-MS2/PP7 (MCP-EGFP for labeling Chr3q29_1 and PCP-mCherry for labeling Chr3q29_2) and (**D**) CRISPR/MB (ATTO647N-conjugated anti-MTS*β* MB for labeling Chr3q29_1 and ATTO550-conjugated anti-MTS*α* MB for labeling Chr3q29_2) imaging systems and used to create the corresponding excised cassettes. Following nucleofection of the constructs into cells containing the necessary imaging components, cells were imaged to look for the presence of colocalized spots and uncolocalized spots (see “Materials and methods”). Representative maximum intensity projection images and both % cells with colocalized spot(s) and % cells with uncolocalized spot(s) are shown. Arrows point to uncolocalized spots. *N* = 6 replicate experiments for CRISPR-MS2/PP7 and 5 replicate experiments for CRISPR/MB, with at least 20 cells examined per experiment. All data represent mean ± SEM. Asterisks indicate significant differences from the plasmid samples (*** *P* < 0.001, ** *P* < 0.01). n.s., not significant. Scale bar, 10 μm.

Given plasmid backbones contribute to the generation of cryptic transcripts, we hypothesized that transfecting only the U6-sgRNA cassette (i.e. sgRNA transcription unit) could lead to reduced cryptic RNA foci formation. To test this, we obtained U6-sgNonsense cassettes by either restriction enzyme digestion or PCR from the parental pUC19-U6-sgNonsense plasmids (Fig. 5A). This was followed by nucleofecting 0.45 pmol of the cassettes or the parental plasmids into RPE-1 cells, a nucleofection condition that could result in comparable intracellular quantities of U6-sgNonsense DNA elements, manifested by similar integrated fluorescence intensities assessed using CASFISH (Supplementary Fig. S7). Following RNA FISH experiments, an average number of 0.09 ± 0.05 foci per excised cassette-transfected cell and 0.16 ± 0.13 foci per PCR-derived cassette-transfected cell were observed (Fig. 5B), in stark contrast to an average foci number of 15.80 ± 1.01 per plasmid-transfected cell (Fig. 1B). Collectively, our findings indicated that when plasmid regions outside of the U6-sgRNA cassette are removed, production of cryptic plasmid transcripts is inhibited, and coalescence of the cryptic transcripts into false-positive foci is impeded.

### U6-sgRNA cassettes confer more accurate genomic labeling than their parental plasmids

Based on the findings that U6-sgRNA cassette transfection may reduce false-positive foci formation compared to transfection of the parental plasmid, we next investigate whether this cassette transfection approach could also improve the accuracy of genomic loci labeling. To test this, we generated a pair of locus-targeting MS2-tagged and PP7-tagged sgRNA expression plasmids with different spacer sequences complementary to distinct repetitive loci within the same genomic region (termed Chr3q29_1 and Chr3q29_2). The corresponding U6-sgRNA cassettes were obtained by either restriction enzyme digestion or PCR. The MS2-tagged and PP7-tagged plasmids, or cassettes, were co-transfected in a 1:1 molar ratio into RPE-1 cells that also expressed dCas9 and the effector probes MCP-EGFP and PCP-mCherry (i.e. CRISPR-MS2/PP7). At 24–48-h post-transfection, dual-color live-cell fluorescence imaging experiments were performed, and results were used to determine the extents of co-labeling and mislabeling on a per cell basis by calculating % cells with colocalized spot(s) and uncolocalized spot(s), respectively (see “Materials and methods”). Since the two different sgRNAs were designed to target the same genomic region, we expected accurate co-labeling if EGFP and mCherry signals both appear as bright spots and if all of the detected EGFP and mCherry spots perfectly colocalize.

It was found that, regardless of the expression system used, discrete EGFP and mCherry bright spots were readily detected in the nucleus. Nearly 100% cells were observed to contain colocalized spot(s) for both the cassette-transfected and plasmid-transfected cells, as expected if both sgRNA expression systems can transcribe functional sgRNAs. Notably, mislabeling was significantly less for the cassette-transfected cells (∼9–12%) than the plasmid-transfected cells (∼58%) (Fig. 5C), with no significant difference detected between the excised or PCR-derived U6-sgRNA cassettes in these measurements. Moreover, the EGFP and mCherry spots that did not colocalize with each other were observed to instead colocalize with the paraspeckle protein PSPC1 (Supplementary Fig. S8), as expected since PSPC1 participates in false-positive foci formation (Fig. 3A and B, Supplementary Fig. S6, and Supplementary Movie S2). More reliable use of the expression cassettes, irrespective of the procurement method (restriction enzyme digestion or PCR), was also observed when analogous experiments were performed with a second CRISPR imaging system termed CRISPR/molecular beacon (i.e. CRISPR/MB), in which antisense oligonucleotide probes (i.e. molecular beacons) were used as effector probes [28] (Fig. 5D). It should be noted that more instances of mislabeling were detected for the plasmid-based imaging systems likely because the plasmid backbones can generate various cryptic plasmid transcripts that may harbor portions of sgRNAs (either full-length or partial) that can be bound by effector probes. Together, these results demonstrated that U6-sgRNA cassettes derived via either restriction enzyme digestion or PCR are equally effective in achieving more accurate genomic imaging compared to their parental plasmids when using CRISPR-MS2/PP7 or CRISPR/MB.

## Discussion

In summary, we showed that when plasmids are used to generate CRISPR sgRNAs in cells, false-positive foci may form and appear indistinguishable from authentic genomic foci when detected by fluorescence imaging. These foci are enriched in plasmid-derived cryptic RNA transcripts rather than the intended sgRNAs themselves, regardless of sgRNA scaffold design (Figs 1 and 2 and Supplementary Figs S1–S4). Their formation also requires paraspeckle core proteins such as FUS and SFPQ, but not NEAT1_2, the major RNA scaffold of paraspeckles, and can potentially occur via LLPS (Figs 3 and 4 and Supplementary Fig. S6). Notably, transfecting U6-sgRNA cassettes derived from either restriction enzyme digestion or PCR, which should also continuously transcribe sgRNAs, led to marginal levels of false positives compared to transfecting their parental plasmids (Fig. 5B). This confirms the dependence of false-positive foci formation on cryptic plasmid transcripts. We further demonstrated that while using U6-sgRNA cassettes and the parental plasmids may both lead to similar co-labeling efficiency (∼100%), the former yielded significantly less mislabeling (5–12%) than the latter (35–58%) (Fig. 5C and D). Considering the often diploid or triploid nature of many primary cells and cell lines of mammalian origin, the observed differences in mislabeling should underscore the importance of choosing sgRNA transcription units (e.g. U6-sgRNA cassette) over their parental plasmids for CRISPR-mediated genomic imaging tools that use fluorescently tagged sgRNAs. Additionally, the findings also suggest that in other reporter assays (such as RNA imaging) where RNAs of interest are transiently expressed from their DNA templates, the use of promoter-gene cassettes should be considered as an alternative to the parental plasmids since the former can potentially generate fewer false-positive signals. Moreover, our results should also have implications in the analysis of transcription regulations based on reporter-gene assays, such as when the intracellular activities of specific promoters or enhancers are to be quantitated, since plasmid backbones may possess DNA regions that display cryptic transcription activities [16–18, 29, 51].

**Figure 6.**
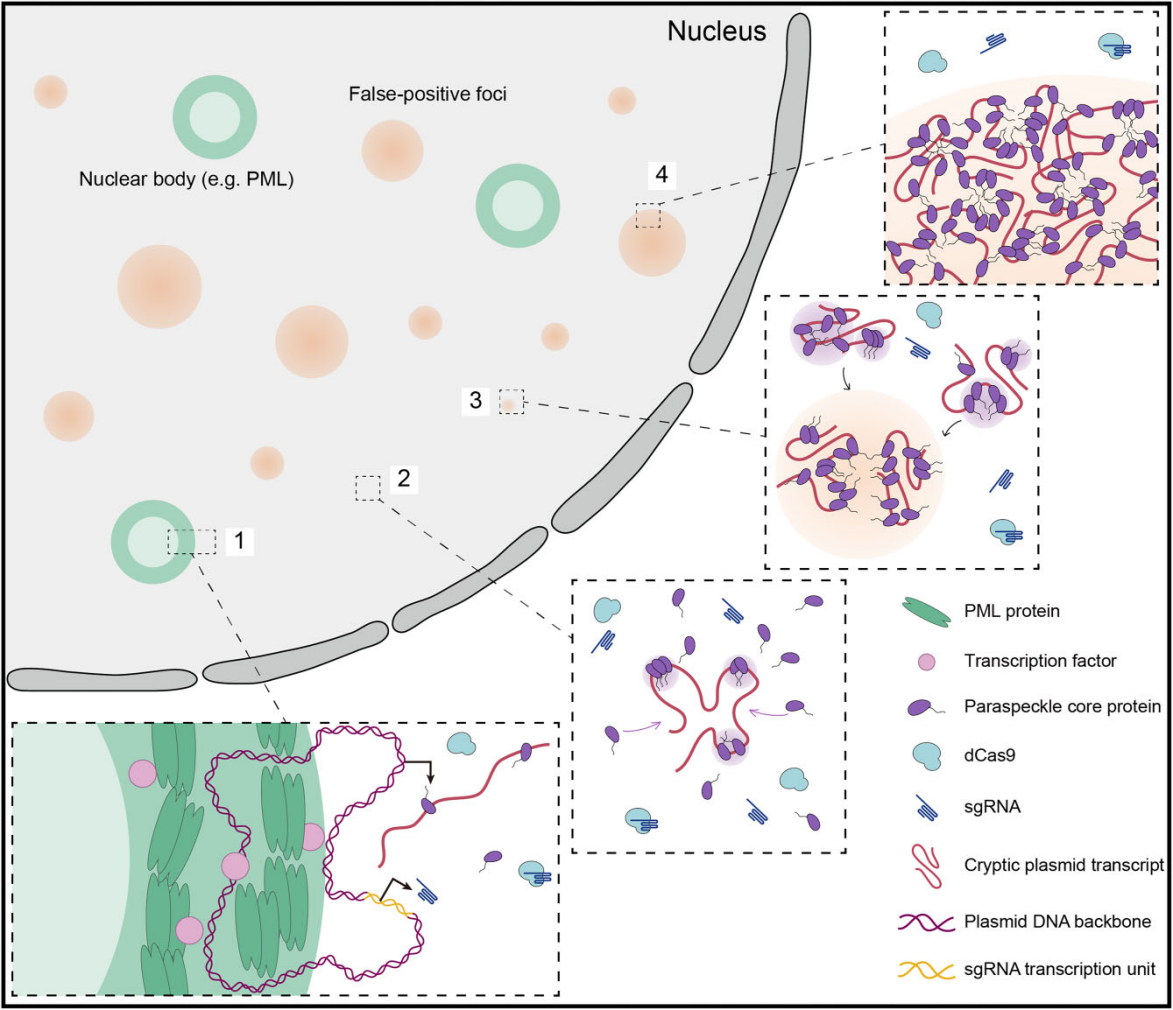
Schematic model of cryptic plasmid transcript accumulation and false-positive foci formation. (**1**) Following transcription, cryptic plasmid transcripts rapidly bind a few paraspeckle core proteins FUS, SFPQ, and PSPC1 to initiate cryptic RNA foci assembly. (**2**) By virtue of these proteins’ phase separation capabilities, their local concentrations on the cryptic transcripts increase, which in turn, attract more paraspeckle core proteins to bind cryptic RNAs. (**3–4**) Coalescence of multiple paraspeckle core protein-associated cryptic transcripts leads to formation of larger false-positive foci that display liquid-like properties. This cryptic plasmid transcript-scaffolded protein assembly process does not appear to affect the transcription of the intended sgRNAs from sgRNA transcription units on plasmids.

To date, the majority of the cryptic plasmid transcription-relevant studies have focused on identifying the origin of the unintended activity and have relied on lysate-based biochemical assays. This study, to our knowledge, is the first study that investigates the subcellular localizations of cryptic plasmid transcripts and their biophysical properties by cellular and molecular fluorescence imaging. In particular, our findings with sgRNA producer plasmids uncovered the potential role of cryptic plasmid transcripts in scaffolding the assembly of paraspeckle core proteins (e.g. FUS, SFPQ, and PSPC1) into liquid droplet-like biomolecular condensates without the involvement of the producer plasmids or the paraspeckle's major RNA component NEAT1_2 (Figs 3 and 4 and Supplementary Fig. S6). Together with studies showing that these paraspeckle proteins display capacities to bind RNA via electrostatic interactions [52–57], we proposed that cryptic plasmid transcripts following transcription rapidly bind the paraspeckle proteins to initiate cryptic RNA foci assembly (Fig. 6). The associated increase in the local concentrations of the paraspeckle proteins, in turn, attract additional paraspeckle proteins, potentially through their phase separation capabilities [45–50], ultimately leading to formation of larger foci. This process does not appear to impact the activities of the sgRNA transcription units on the plasmids, since the intended sgRNAs may still bind and guide dCas9 to authentic genomic loci (as indicated by near ∼100% labeling of authentic genomic loci shown in Fig. 5C and D).

The observed cryptic plasmid transcript-mediated scaffolding of paraspeckle proteins also raise several pivotal questions that could potentially pave new paths in paraspeckle research. For instance, it would be intriguing to understand whether these cryptic transcript- and paraspeckle core protein-enriched nuclear bodies also require other proteins to form and whether they exhibit the characteristic core-shell spheroidal structure observed with canonical paraspeckles as revealed by super-resolution microscopy [58, 59]. In addition, it remains to be determined whether the observed usurps of paraspeckle proteins by cryptic plasmid transcripts may disrupt normal paraspeckle functions, such as gene expression regulation [40, 48, 58, 60]. Furthermore, given that paraspeckles are enriched in adenosine-to-inosine edited RNA molecules [61], it may be intriguing to explore whether the sequestered cryptic transcripts contain certain sequence characteristics, modifications, or secondary structures that can more readily recruit paraspeckle proteins to bind. The findings from such studies may also shed light on the mechanisms driving the formation of previously identified paraspeckle-like structures [62, 63], whose formation is scaffolded by nucleic acids other than NEAT1_2.

## Supplementary Material

gkaf192_Supplemental_Files

## Data Availability

The data underlying this article are available in the article and in its online supplementary material.
