# Supplementary material for: Spuriously transcribed RNAs from CRISPR-sgRNA expression plasmids scaffold biomolecular condensate formation and hamper accurate genomic imaging
Source: Nucleic Acids Res. 2025 Mar 22;53(6):gkaf192. doi: 10.1093/nar/gkaf192 (PMC11928936; doi:10.1093/nar/gkaf192)
Supplement: gkaf192_Supplemental_Files [file gkaf192_supplemental_files.zip › MSQ WRN et al 1212 NAR-03018-Met-G-2024 R1 Supplementary.pdf]

Supplementary Table S1. Sequences of the sgRNA scaffolds used in this study.

**sgRNA scaffold name**	**sgRNA scaffold sequence**
sgRNA-Original	GTTTTAGAGCTAGAAATAGCAAGTTAAAATAAGGCTAGTCCGTT ATCAACTTGAAAAAGTGGCACCGAGTCGGTGCTTTTTTT
sgRNA-2XMS2	GTTTGAGAGCTAGGCCAACATGAGGATCACCCATGTCTGCAGGG CCTAGCAAGTTCAAATAAGGCTAGTCCGTTATCAACTTGGCCAA CATGAGGATCACCCATGTCTGCAGGGCCAAGTGGCACCGAGTCG GTGCTTTTTTT
sgRNA-2XPP7	GTTTGAGAGCTACCGGAGCAGACGATATGGCGTCGCTCCGGTAG CAAGTTCAAATAAGGCTAGTCCGTTATCAACTTGGAGCAGACGA TATGGCGTCGCTCCAAGTGGCACCGAGTCGGTGCTTTTTTT
sgRNA-MTS*α*	GTTTGAGAGCTATGCTGGAAACAGCATAGCAAGTTCAAATAAGG CTAGTCCGTTATCAACTTGGCCCCGGAGCAGAACGACTCAGTCA CGACATCACTTACGCTAGCCTGCAGTCTGCTCCGGGGCCAGTGG CACCGAGTCGGTGCTTTTTTT
sgRNA-MTS*β*	GTTTGAGAGCTATGCTGGAAACAGCATAGCAAGTTCAAATAAGG CTAGTCCGTTATCAACTTGGCCCCGGAGCAGAACGACAGGAGTT GTGTTTGTGGACGAAGAGCCTGCAGTCTGCTCCGGGGCCAGTGG CACCGAGTCGGTGCTTTTTTT
sgRNA-CASFISH	GTTTAAGAGCTATGCTGGAAACAGCATAGCAAGTTTAAATAAGG CTAGTCCGTTATCAACTTGAAAAAGTGGCACCGAGTCGGTGCTTT TTTT

Supplementary Table S2. Spacer sequence and target region information of the sgRNAs used in this study.

sgRNA name	sgRNA scaffold name	Target region				sgRNA spacer sequence
		Chromosome number	Copy number	Position		
				Start	End	
sgNonsense	sgRNA-Original	N.A.	N.A.	N.A.	N.A.	GGAGTTGTGTTTG TGGACGAAG
sgNonsense_2	sgRNA-Original	N.A.	N.A.	N.A.	N.A.	GTCACGACATCAC TTACGCTGA
sgNonsense-2XMS2	sgRNA-2XMS2	N.A.	N.A.	N.A.	N.A.	GGAGTTGTGTTTG TGGACGAAG
sgChr3q29_1-2XMS2	sgRNA-2XMS2	3	101	195,478, 315	195,506, 987	GCTCCTCTGTATG ATATCACAG
sgChr3q29_2-2XPP7	sgRNA-2XPP7	3	124	195,478, 353	195,506, 949	GCTCCTCTGTGTG ATATCACAG
sgChr3q29_1-MTSβ	sgRNA-MTSβ	3	101	195,478, 315	195,506, 987	GCTCCTCTGTATG ATATCACAG
sgNonsense-MTSα	sgRNA-MTSα	N.A.	N.A.	N.A.	N.A.	GGAGTTGTGTTTG TGGACGAAG
sgChr3q29_2-MTSα	sgRNA-MTSα	3	124	195,478, 353	195,506, 949	GCTCCTCTGTGTG ATATCACAG
sgNonsense_IVT	sgRNA-Original	N.A.	N.A.	N.A.	N.A.	GGGGGAGTTGTGT TTGTGGACGAAG
sgNonsense-2XMS2_IVT	sgRNA-2XMS2	N.A.	N.A.	N.A.	N.A.	GGGGGAGTTGTGT TTGTGGACGAAG
sgNonsense-MTSα_IVT	sgRNA-MTSα	N.A.	N.A.	N.A.	N.A.	GGGGGAGTTGTGT TTGTGGACGAAG
sgCASFISH	sgRNA-CASFISH	N.A.	N.A.	N.A.	N.A.	GGGCTTGAAAAA GTGGCACCGAGT

Supplementary Table S3. Sequences of PCR primers used for constructing sgRNA expression plasmids by PCR-mediated site-directed mutagenesis.

sgRNA name	Forward primers	Reverse primers
sgNonsense_2	GTCACGACATCACTTACGCTGAGTT TTAGAGCTAGAAATAGCAAGTT	CAACAAGGTGGTTCTC CAAGGGATA
sgChr3q29_1-2XMS2	GCTCCTCTGTATGATATCACAGGTTT GAGAGCTAGGCCAACA	CAACAAGGTGGTTCTC CAAGGGATA
sgChr3q29_2-MTSα	GCTCCTCTGTGTGATATCACAGGTTT GAGAGCTATGCTGGAAACA	CAACAAGGTGGTTCTC CAAGGGATA

Supplementary Table S4. Sequences and flurophore tags of the RNA FISH probes used to label nonsense sequences and cryptic plasmid transcripts

**Name**	**Sequence**	**Fluorophore**
anti-Nonsense	5’-CTTCGTCCACAAACACAACTCCTG-3’	ATTO647N
anti-Nonsense_2	5’-CTCAGCGTAAGTGATGTCGTGAC-3’	ATTO550
anti-CPT_1	5’-AACGACCTACACCGAACTGAGA-3’	ATTO647N or Alexa Fluor^{TM} 488
anti-CPT_2	5’-TGTTGTGTGGAATTGTGAGCGG-3’	ATTO647N

Supplementary Table S5. Information and usage of the primary antibodies used in this study. IF, immunofluorescence; WB, western blot; RT, Room temperature.

Target	Antibody	Source	Identifier	Antibody dilution buffer	Dilution ratio	Incubation temperature	Incubation time
TRIM19/ PML	Mouse anti-PML (for IF)	MBL Life Science	M041-3	2% (vol/vol) FBS in 1x PBS	1:1000	RT	30 min
SC35	Mouse anti-SC35 (for IF)	Santa Cruz	sc-53518	0.1% (wt/vol) BSA in 1x PBS	1:1000	RT	1 h
FUS	Mouse anti-FUS/TLS (for IF)	Santa Cruz	sc-47711	0.1% (wt/vol) BSA in 1x PBS	1:1000	RT	1 h
SFPQ	Mouse anti-SFPQ (for IF)	MBL Life Science	RN014MW	2% FBS (vol/vol) in 1x PBS	1:2000	RT	30 min
PSPC1	Mouse anti-PSPC1 (for IF)	MBL Life Science	RN015MW	2% FBS (vol/vol) in 1x PBS	1:2000	RT	30 min
hnRNP A1	Mouse anti-hnRNP A1 (for IF)	Santa Cruz	sc-32301	0.1% (wt/vol) BSA in 1x PBS	1:1000	RT	1 h
hnRNP U	Mouse anti-hnRNP U (for IF)	Santa Cruz	sc-32315	0.1% (wt/vol) BSA in 1x PBS	1:1000	RT	1 h
TDP-43	Rabbit anti-TDP-43 (for IF)	Proteintech	10782-2-AP	0.1% (wt/vol) BSA in 1x PBS	1:1000	RT	1 h
FUS	Rabbit anti-FUS/TLS(fo r WB)	Proteintech	11570-1-AP	1x TBS, 0.05% (vol/vol) Tween-20 with 5% (wt/vol) nonfat dry milk	1:5000	4ºC	overnight

SFPQ	Mouse anti-SFPQ (for WB)	MBL Life Science	RN014MW	1x TBS, 0.05% (vol/vol) Tween-20 with 5% (wt/vol) nonfat dry milk	1:5000	4ºC	overnight
GAPDH	Mouse anti-GAPDH (for WB)	Proteintech	60004-1-Ig	1x TBS, 0.05% (vol/vol) Tween-20 with 5% (wt/vol) nonfat dry milk	1:500000	RT	1 h

Supplementary Figure S1.

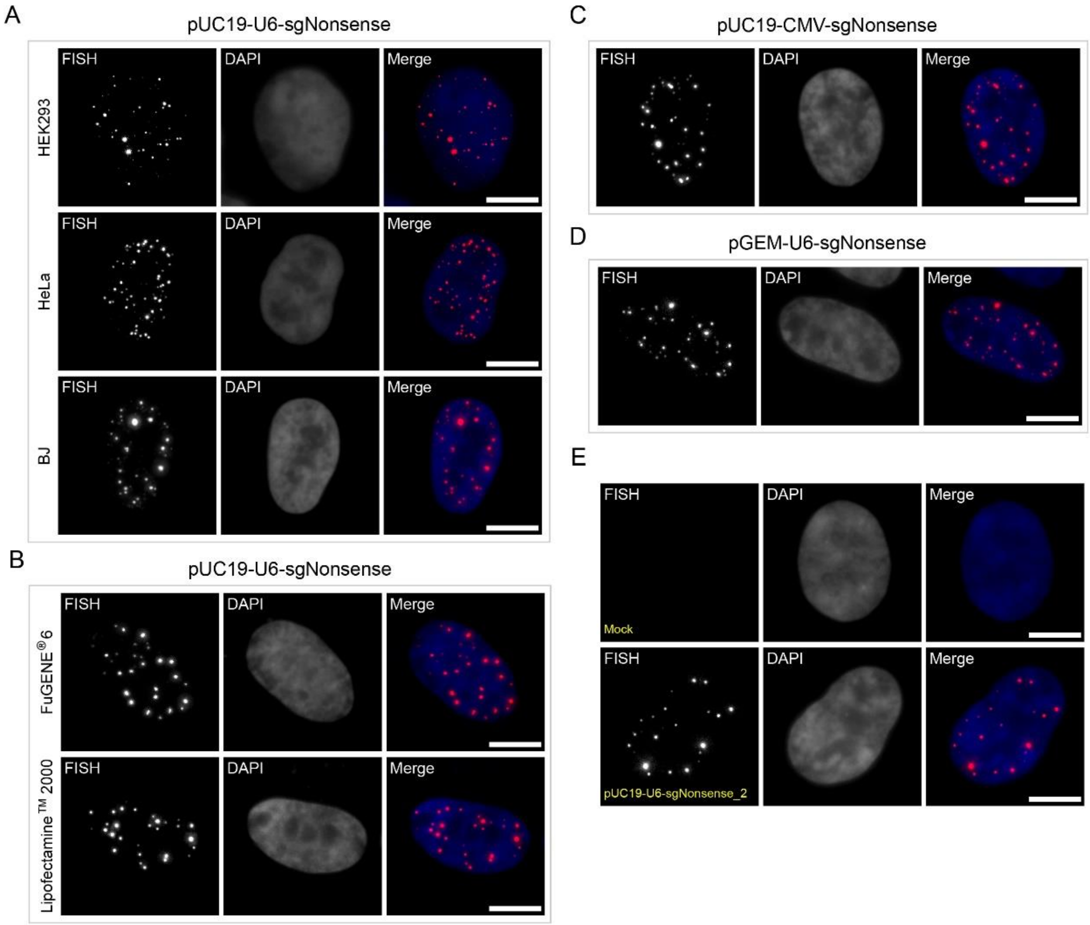

Figure S1. Transfecting sgRNA-expression plasmids leads to false-positive foci in cell nuclei regardless of cell type, transfection method, promoter type, vector type or nonsense sequence. (A-D) RNA FISH was performed using the anti-Nonsense probe at 24 h post-transfection in (**A**) HEK293, HeLa or BJ cells nucleofected with 0.45 pmol of pUC19-U6-sgNonsense, (**B**) hTERT RPE-1 (RPE-1) cells transfected with 0.13 pmol of pUC19-U6-sgNonsense by either FuGENE® 6 or Lipofectamine™ 2000, (**C**) RPE-1 cells nucleofected with 0.45 pmol of pUC19-CMV-sgNonsense, aand (**D**) RPE-1 cells nucleofected with 0.45 pmol of pGEM-U6-sgNonsense. (**E**) RNA FISH was performed using the anti-Nonsense_2 probe at 24 h post-transfection in RPE-1 cells mock nucleofected or nucleofected with 0.45 pmol of pUC19-U6-sgNonsense_2. Representative maximum intensity projection images are shown. DAPI stains the nucleus. Scale bar, 10 µm.

Supplementary Figure S2.

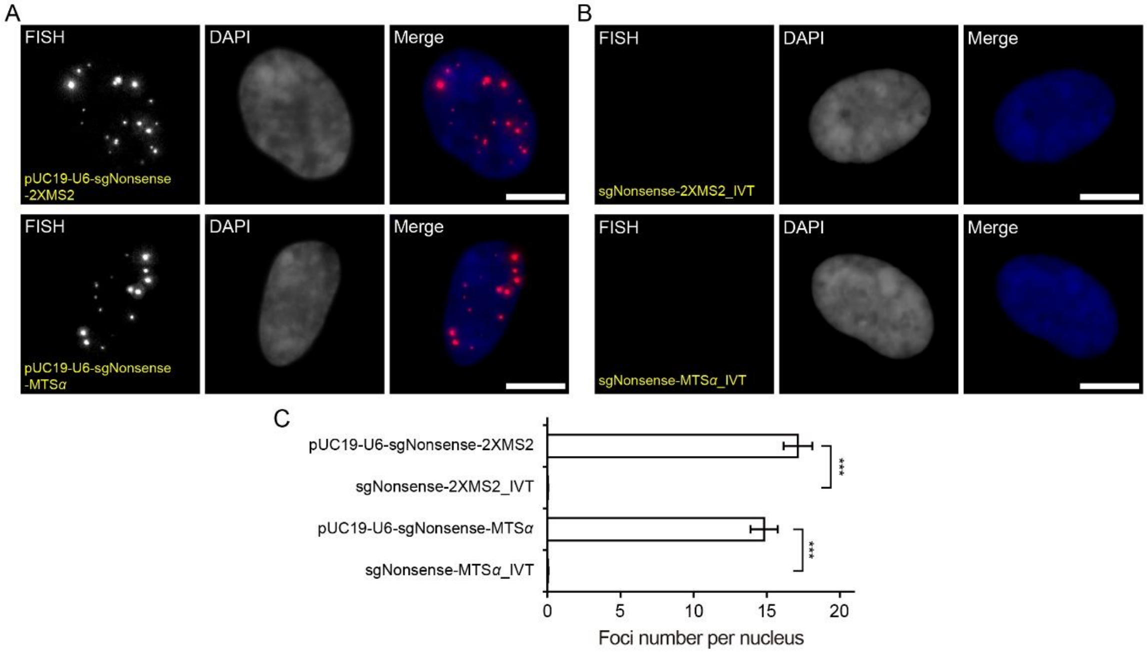

Figure S2. Transfecting modified sgRNA-expression plasmids, but not modified sgRNAs, results in false-positive foci formation. (**A**) RPE-1 cells were nucleofected with 0.45 pmol of the plasmid encoding modified sgNonsense that incorporates the MS2 aptamer (pUC19-U6-sgNonsense-2XMS2) or the plasmid encoding modified sgNonsense that incorporates the MB target sequence (pUC19-U6-sgNonsense-MTS*α*). The cells were subjected to RNA FISH using the anti-Nonsense probe at 24 h post-nucleofection. Representative maximum intensity projection images are shown. (**B**) RPE-1 cells were nucleofected with 4 µM of *in vitro*-transcribed sgRNAs, sgNonsense-2XMS2_IVT or sgNonsense-MTS*α*_IVT. The cells were subjected to RNA FISH using the anti-Nonsense probe at 24 h post-nucleofection. Representative maximum intensity projection images are shown. (**C**) Average numbers of FISH foci detected in cells from (A-B). Data represent mean ± SEM of 70 pUC19-U6-sgNonsense-2XMS2-transfected cells, 59 sgNonsense-2XMS2_IVT-transfected cells, 73 pUC19-U6-sgNonsense-MTS*α*-transfected cells, and 68 sgNonsense-MTS*α*_IVT-transfected cells. Asterisks indicate significant differences (*** $P < 0.001$). DAPI stains the nucleus. Scale bar, 10 µm.

Supplementary Figure S3.

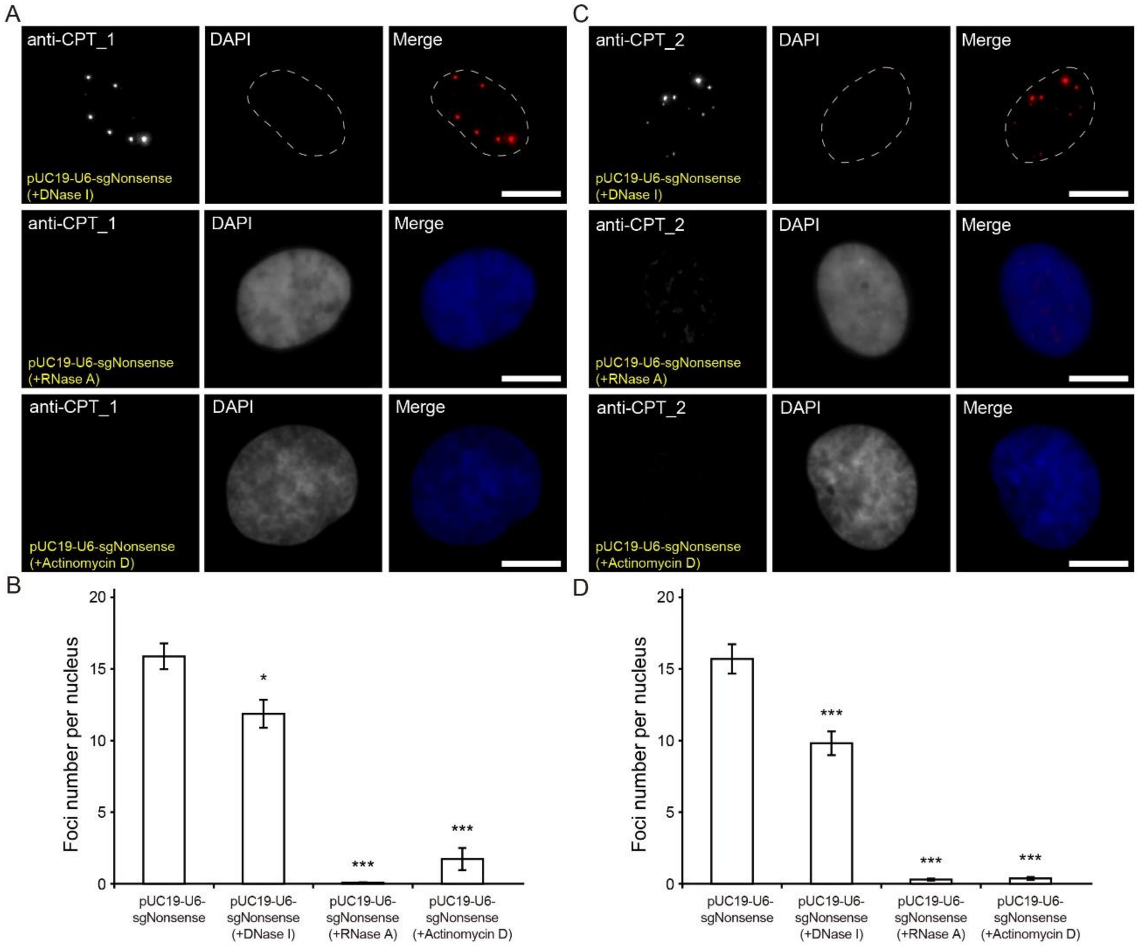

Figure S3. Treatment with RNase A or actinomycin D, but not with DNase I, significantly disrupted nonspecific foci detected by RNA FISH using the anti-cryptic plasmid transcript (CPT) probes. RPE-1 cells were nucleofected with 0.45 pmol of pUC19-U6-sgNonsense. The cells were further treated with DNase I, RNase A or actinomycin D and then subjected to RNA FISH using ATTO647N-conjuggated anti-CPT_1 or anti-CPT_2 probes. Note that cell fixation and permeabilization was performed before the DNase I and RNase A treatments and after the actinomycin D treatment. (**A**) Representative maximum intensity projection images of RNA FISH using the anti-CPT_1 probe. (**B**) Average numbers of FISH foci detected in cells from (A), plotted together with the average number of FISH foci detected by the anti-CPT_1 probe in (untreated) pUC19-U6-sgNonsense-transfected cells from Figure 2A. N= 67, 45, 45, and 39 cells for the pUC19-U6-sgNonsense, pUC19-U6-sgNonsense (+DNase I), pUC19-U6-sgNonsense (+RNase A), and pUC19-U6-sgNonsense (+Actinomycin D) samples, respectively.(**C**) Representative maximum intensity projection images of RNA FISH using the anti-CPT_2 probe. (**D**) Average numbers of FISH foci detected in cells from (C), plotted together with the average number of FISH foci detected by the anti-CPT_2 probe in (untreated) pUC19-U6-sgNonsense-transfected cells from Figure 2A. N= 77, 47, 69, and 46 cells for the pUC19-U6-sgNonsense, pUC19-U6-sgNonsense (+DNase I), pUC19-U6-sgNonsense (+RNase A), and pUC19-U6-sgNonsense (+Actinomycin D) samples, respectively. The nucleus is indicated either by DAPI staining or dashed line (in the DNase I-treated cells).

All data represent mean ± SEM. Asterisks indicate significant differences from pUC19-U6-sgNonsense-transfected cells (* $P < 0.05$, *** $P < 0.001$). Scale bar, 10 μm.

Supplementary Figure S4.

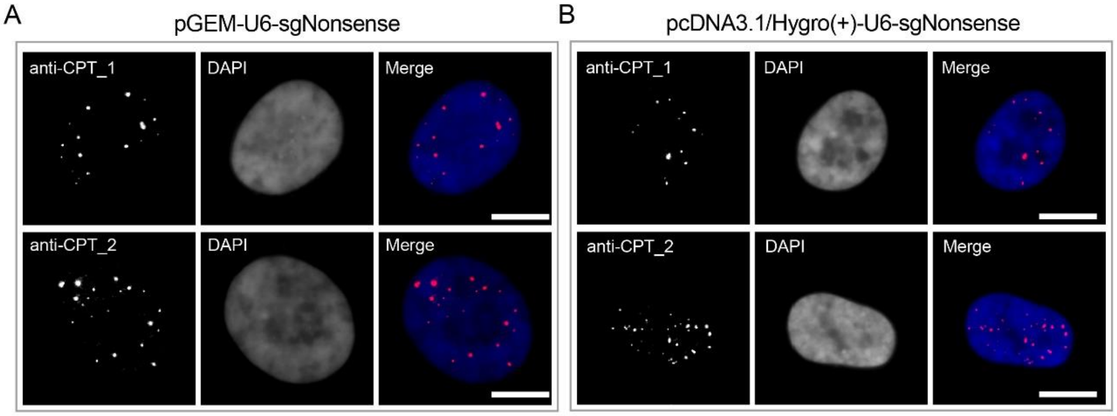

Figure S4. Cryptic transcripts from different sgRNA-expression plasmids form false-positive foci. RPE-1 cells were nucleofected with 0.45 pmol of pGEM-U6-sgNonsense or 0.45 pmol of pcDNA3.1/Hygro(+)-U6-sgNonsense. At 24 h post-nucleofection, the cells were subjected to RNA FISH using ATTO647N-conjugated anti-CPT_1 or anti-CPT_2 probes. Representative maximum intensity projection images are shown. DAPI stains the nucleus. Scale bar, 10 µm.

Supplementary Figure S5.

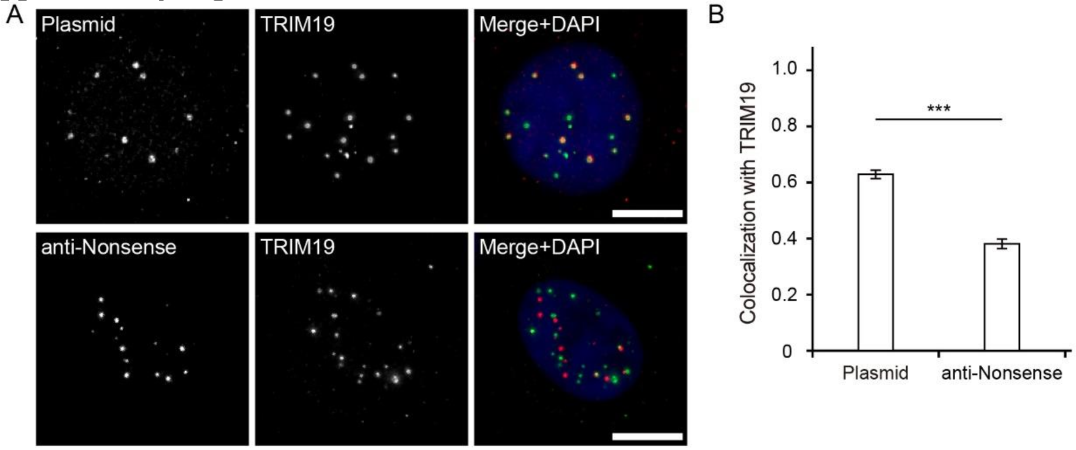

Figure S5. pUC19-U6-sgNonsense plasmid DNA-positive foci, but not anti-Nonsense-positive RNA foci, colocalize with TRIM19/PML. RPE-1 cells were nucleofected with 0.45 pmol of pUC19-U6-sgNonsense. At 24 h post-nucleofection, the cells were subjected to CASFISH or RNA FISH in combination with immunofluorescence using anti-PML antibodies. (**A**) Representative maximum intensity projection images. DAPI stains the nucleus. Scale bar, 10 µm. (**B**) Colocalization (Pearson's correlation coefficients) between TRIM19/PML and plasmid signals (n=34 cells) or between TRIM19/PML and anti-Nonsense signals (n=29 cells) detected in cells from (A). Data represent mean ± SEM. Asterisk indicates significant difference (*** $P < 0.001$).

Supplementary Figure S6.

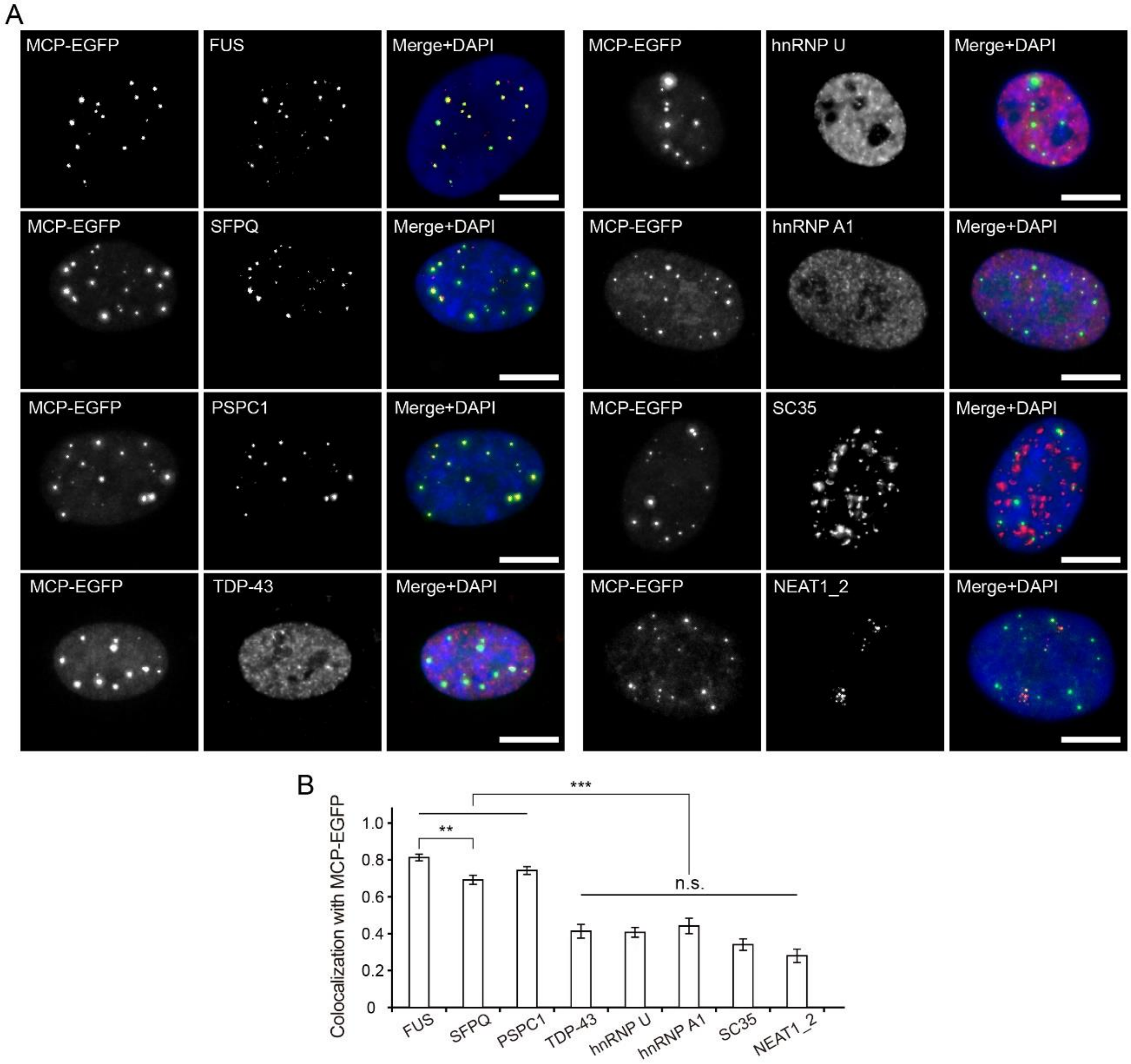

Figure S6. Colocalizations between false-positive foci detected by MCP-EGFP and candidate RNA binding proteins or NEAT1_2 RNA. RPE-1 cells were nucleofected with 20 ng of MS2_EGFP and 867 ng (i.e., 0.45 pmol) of pUC19-U6-sgNonsense-2XMS2. At 24 h post-nucleofection, cells were subjected to immunofluorescence for the indicated proteins (Alexa Fluor^{TM} 647-conjugated secondary antibodies) or RNA FISH for NEAT1_2 (Quasar® 570-labeled). (**A**) Representative maximum intensity projection images. DAPI stains the nucleus. Scale bar, 10 µm. (**B**) Colocalization (Pearson's correlation coefficients) between MCP-EGFP signals and fluorescence signals of the indicated nuclear markers. N= 33, 20, 24, 23, 22, 20, 21 and 16 cells for the studies with FUS, SFPQ, PSPC1, TDP-43, hnRNP U, hnRNP A1, SC35, and NEAT1_2, respectively. Note that the extents of colocalization were in general lower than the results obtained with the FISH experiments shown in Figure 3, partly because free and unbound MCP-EGFP could contribute substantially to background fluorescence, which can lead to underestimation of colocalization assessed by Pearson's correlation coefficients. Data

represent mean ± SEM. Asterisks indicate significant differences (*** $P < 0.001$, ** $P < 0.01$). n.s., not significant.

Supplementary Figure S7.

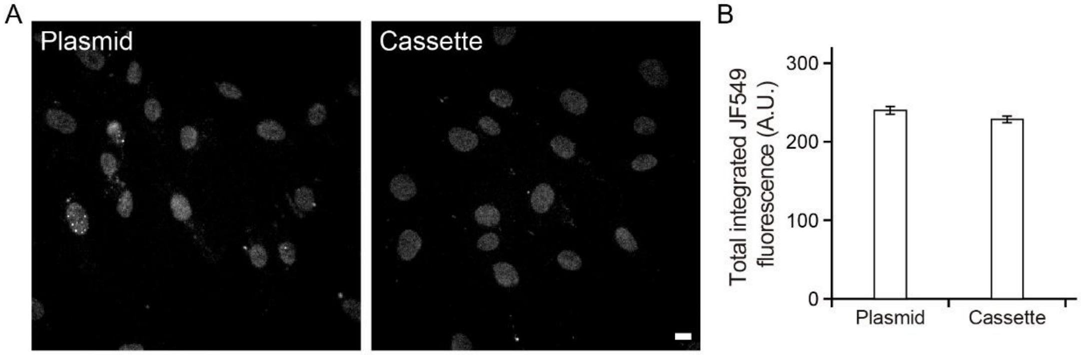

Figure S7. CASFISH reveals similar intracellular quantities of the sgRNA encoding DNA region resulting from nucleofecting 0.45 pmol of pUC19-U6-sgNonsense or of the excised U6-sgNonsense cassette into RPE-1 cells. At 24 h post-nucleofection, the cells were subjected to CASFISH. (**A**) Representative images. Scale bar, 10 µm. (**B**) Total integrated JF549 fluorescence detected in cells from (A). Data represent mean ± SEM of 150 cells for each sample. No difference was detected. A.U., arbitrary unit.

Supplementary Figure S8.

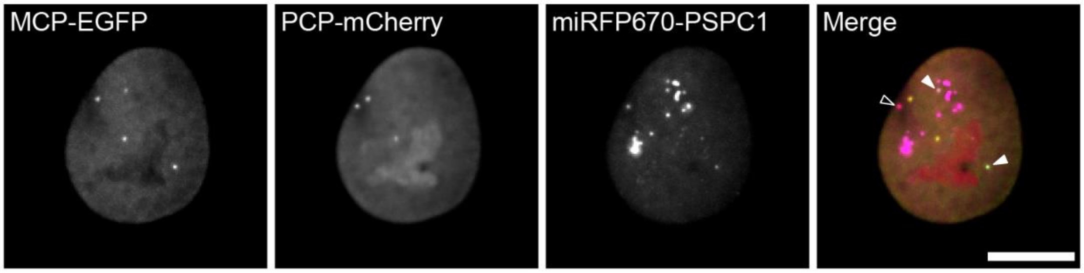

Figure S8. CRISPR-MS2/PP7 can nonspecifically colocalize with PSPC1. RPE-1 cells were nucleofected with 150 ng of pdCas9, 20 ng of MS2_EGFP, 20 ng of PP7_mCherry, 865 ng (i.e., 0.45 pmol) of pUC19-U6-sgChr3q29_1-2XMS2 plus pUC19-U6-sgChr3q29_2-2XPP7 (at a 1:1 molar ratio), and 20 ng of miRFP670-PSPC1. Representative maximum intensity projection images acquired at 24 h post-nucleofection are shown. The solid arrows point to MCP and PSPC1 colocalized spots and the open arrow points to a PCP and PSPC1 colocalized spot. Scale bar, 10 µm.

Supplementary Movie S1 (separate file). A representative movie of nonspecific foci formed in live cells transfected with sgNonsense-2XMS2 expressing plasmids. RPE-1 cells were nucleofected with 20 ng of MS2_EGFP and 867 ng (i.e., 0.45 pmol) of pUC19-U6-sgNonsense-2XMS2, and imaged at 48 h post-nucleofection. The acquisition rate was 10 frames per second. The video includes 200 frames and the play rate is 10 frames per second. Note that nonspecific foci are in general slow moving and sparsely distributed in space. Scale bar, 10 µm.

Supplementary Movie S2 (separate file). A representative movie showing colocalization and co-movement of PSPC1 and nonspecific foci formed in live cells transfected with sgNonsense-2XMS2 expressing plasmids. RPE-1 cells were nucleofected with 20 ng of MS2_EGFP (pseudocolored green), 867 ng (i.e., 0.45 pmol) of pUC19-U6-sgNonsense-2XMS2, and 20 ng of miRFP670-PSPC1 (pseudocolored red). The cells were imaged at 48 h post-nucleofection. The acquisition rate was 10 frames per second. The video includes 199 frames and the play rate is 10 frames per second. Scale bar, 10 µm.
